# Pharmacogenomics of Sorafenib in Hepatocellular Carcinoma (HCC): A LncRNA-Expression Guided Approach Using UCA1 and MALAT1 for Personalizing Therapy in a 154-Patient Cohort

**DOI:** 10.3390/ph19010070

**Published:** 2025-12-29

**Authors:** Mahmoud Nazih, Imam Waked, Shimaa Abdelsattar, Hiba S. Al-Amodi, Hala F. M. Kamel, Muhammad Mahmoud Attia, Ahmed I. Khoder, Sahar Badr Hassan, Mohamed Mahmoud Abdel-Latif

**Affiliations:** 1 Department of Clinical Pharmacy, Faculty of Pharmacy, Assiut University, Assiut 71526, Egypt; 2 Department of Clinical Pharmacology, Al Ryada University for Science and Technology (RST), ElMehwar ElMarkazy-2, Cairo—Alex desert RD K92, Sadat City 16504, Egypt; 3 Scientific Office, Egyptian Society of Pharmacogenomics and Personalized Medicine (ESPM), Cairo 11562, Egypt; 4 Hepatology and Gastroenterology Department, National Liver Institute (NLI), Menoufia University, Shebin El-Kom 32511, Egypt; 5 Clinical Biochemistry and Molecular Diagnostics, National Liver Institute (NLI), Menoufia University, Shebin El-Kom 35211, Egypt; 6 Biochemistry Department, Faculty of Medicine, Umm Al-Qura University, Makkah 21955, Saudi Arabia; 7 Medical Biochemistry and Molecular Biology Department, Faculty of Medicine, Ain Shams University, Cairo 11566, Egypt; 8 Software Engineer, Cairo 11837, Egypt

**Keywords:** hepatocellular carcinoma (HCC), sorafenib, lncRNA, UCA1, MALAT1, drug resistance, personalized therapy, pharmacogenomics

## Abstract

**Background/Objectives**: Hepatocellular carcinoma (HCC) presents limited therapeutic options for advanced disease, and sorafenib therapy is hampered by significant interpatient heterogeneity in response. This necessitates biomarker-guided strategies to personalize treatment. This study investigated the long noncoding RNAs UCA1 and MALAT1 as pharmacogenomic biomarkers for personalizing sorafenib therapy in advanced HCC. **Methods**: In a prospective cohort of 154 HCC patients receiving first-line sorafenib (400 mg twice daily), serum lncRNA levels were quantified by RT-qPCR at baseline, Week 4, and Week 12. Expression levels were correlated with treatment response (mRECIST), time-to-progression (TTP), and overall survival (OS). Statistical analyses included Kaplan–Meier estimates, Cox proportional hazards models, and ROC curve analysis. **Results**: High baseline expression of UCA1 (77.9% of patients) and MALAT1 (73.4%) was associated with aggressive disease. High UCA1 correlated with reduced 12-month survival (60.8% vs. 73.5%, *p* = 0.026) and shorter median Time-to-Progression (TTP) (18.0 vs. 21.9 weeks, *p* = 0.002). High MALAT1 was associated with significantly shorter median TTP (18.0 vs. 25.2 weeks, *p* = 0.003). In multivariable analysis, both lncRNAs were independent prognostic factors for shorter TTP (UCA1: HR = 1.52, *p* = 0.014; MALAT1: HR = 1.61, *p* = 0.006). Serial monitoring revealed that a ≥10% rise in either lncRNA by Week 4 predicted a five-fold higher progression risk by Week 12 (52% vs. 10%, *p* < 0.001), providing a median lead time of 7.0 weeks before radiological confirmation of progression. **Conclusions:** These findings demonstrate that UCA1 and MALAT1 enable early identification of sorafenib resistance. Baseline stratification and serial monitoring can provide early detection of treatment resistance, informing clinical decision-making and supporting their potential utility for personalizing therapy in advanced HCC.

## 1. Introduction

Hepatocellular carcinoma (HCC) stands as a formidable global health challenge, ranking as the third leading cause of cancer-related mortality worldwide [1]. Its pathogenesis is often insidious, with a majority of patients presenting with advanced, inoperable disease, where therapeutic options have historically been limited and prognosis dismal [2]. For decades, advanced HCC was considered a chemo-resistant malignancy, creating a pressing need for novel therapeutic strategies that could meaningfully improve patient survival.

The turn of the millennium heralded a new era in HCC management with the advent of molecularly targeted therapies. The development and subsequent approval of sorafenib, a multikinase inhibitor, represented a paradigm shift, establishing the first systemic treatment proven to significantly improve overall survival in patients with advanced HCC and ending a long period of therapeutic stagnation [3]. Sorafenib exerts its effects by targeting a constellation of serine-threonine and tyrosine kinases involved in tumor proliferation (Raf/MEK/ERK pathway) and angiogenesis (VEGFR, PDGFR), thereby impeding both tumor growth and its blood supply [4].

This breakthrough cemented sorafenib’s position as a first-line standard of care, a status reaffirmed in contemporary international guidelines [5,6]. However, the initial optimism has been tempered by a stark clinical reality: the benefits of sorafenib are neither universal nor durable. A profound interpatient heterogeneity in treatment response is observed, with only a subset of patients deriving significant benefit, while the majority exhibit primary (innate) resistance or invariably develop acquired resistance within six months of therapy initiation [3,7]. This renders a significant proportion of patients susceptible to the drug’s toxicities and financial costs without any survival advantage, underscoring a critical, unresolved “black box” of sorafenib response dynamics.

This clinical enigma has fueled intensive research into the molecular drivers of sorafenib resistance. Beyond established mechanisms involving kinase rewiring and autophagy, the noncoding genome has emerged as a critical regulator of cancer pathogenesis and therapy response [8]. Among these, long noncoding RNAs (lncRNAs) transcripts longer than 200 nucleotides with low protein-coding potential have been implicated as master regulators of gene expression, influencing virtually all hallmarks of cancer, including in HCC [9].

Two lncRNAs, in particular, have garnered significant attention for their roles in HCC aggressiveness: Metastasis-Associated Lung Adenocarcinoma Transcript 1 (MALAT1) and Urothelial Carcinoma-Associated 1 (UCA1). Preclinical models have elucidated their oncogenic functions: MALAT1 promotes HCC cell proliferation, metastasis, and chemoresistance by modulating pathways such as miR-140 sponging and TLR4/NF-κB signaling [10,11], while UCA1 drives tumor growth and confers resistance to various agents by sequestering tumor-suppressive miRNAs, such as miR-216b and miR-138-5p [12,13]. Importantly, their clinical relevance is supported by growing evidence. For instance, Abdelsattar et al. (2025) recently demonstrated that both MALAT1 and UCA1 are highly elevated in the serum of HCC patients, showing diagnostic accuracy for distinguishing HCC from chronic HCV infection, and their high expression was strongly correlated with sorafenib resistance [14].

Furthermore, high circulating UCA1 has been consistently correlated with aggressive tumor characteristics, including large tumor size, vascular invasion, and advanced TNM stage, and has been established as an independent prognostic factor for poor overall survival [15]. Similarly, MALAT1 overexpression in tumor tissue has been identified as a powerful predictor of tumor recurrence after liver transplantation, particularly in patients exceeding the Milan criteria [16]. These studies firmly position UCA1 and MALAT1 as prime candidates for biomarker development.

However, while their diagnostic and prognostic potential is becoming clear, a critical gap remains in translating these findings into a dynamic, clinically actionable framework for personalizing sorafenib therapy. The question of whether these lncRNAs can guide treatment decisions in real time, stratify patients at baseline, and provide an early, liquid biopsy-based signal of emerging resistance remains largely unexplored.

The emergence of lncRNAs as modulators of drug response represents a paradigm shift in oncology pharmacogenomics. Unlike protein-coding genes subject to post-transcriptional and post-translational regulation, lncRNAs can exert rapid, direct effects on cellular phenotypes through diverse mechanisms, including chromatin remodeling, transcriptional regulation, post-transcriptional processing, and competitive endogenous RNA (ceRNA) activity [8]. Their tissue-specific expression patterns and relative stability in circulation make them attractive candidates for liquid biopsy-based monitoring. Recent advances in high-throughput sequencing have revealed that lncRNA dysregulation is not merely a bystander effect of malignant transformation but an active driver of cancer hallmarks, including sustained proliferation, evasion of growth suppression, resistance to cell death, metabolic reprogramming, and, critically, resistance to targeted therapies [9].

In the specific context of HCC, comprehensive profiling studies have identified dozens of aberrantly expressed lncRNAs associated with clinical outcomes. However, translation of these discoveries into clinical practice has been limited by several factors: (1) lack of prospective validation in well-defined therapeutic cohorts, (2) absence of standardized quantification methods suitable for clinical laboratories, and (3) insufficient evidence linking baseline expression to dynamic treatment response trajectories. Early diagnostic studies established that elevated circulating MALAT1 and UCA1 can distinguish HCC from cirrhosis with high accuracy (AUC > 0.90) [14,15], but their theragnostic potential—simultaneously predicting treatment benefit and guiding therapy selection—remained unexplored. The critical question is not merely whether these lncRNAs are elevated in HCC, but whether their levels before treatment initiation can identify patients destined to fail sorafenib, and whether changes during therapy provide early signals of emerging resistance, enabling timely therapeutic intervention.

Therefore, this prospective study was designed to comprehensively evaluate the *theragnostic* value of MALAT1 and UCA1 in a well-defined cohort of sorafenib-treated HCC patients. We hypothesize that a combined approach, utilizing baseline expression for prognostic stratification and serial monitoring for dynamic response assessment, can unlock the full potential of these lncRNAs to optimize treatment personalization, ultimately steering patients toward more effective therapeutic strategies and improving clinical outcomes.

## 2. Results

### 2.1. Patient Cohort Characteristics

A total of 154 patients with advanced hepatocellular carcinoma (HCC) who received first-line sorafenib therapy were prospectively enrolled. The cohort exhibited demographic and clinical characteristics typical of an advanced HCC population, with a mean age of 58.6 ± 11.0 years and a strong male predominance (78.6%). The majority of patients (68.8%) presented with Barcelona Clinic Liver Cancer (BCLC) stage C disease. Hepatic function, as assessed by Child-Pugh classification, was preserved (Class A) in 47.4% of patients, while 37.0% and 15.6% were Class B and C, respectively. Baseline laboratory parameters confirmed significant tumor burden and hepatic dysfunction, with a median alpha-fetoprotein (AFP) level of 54.3 ng/mL and moderate elevations in liver transaminases (Table 1).

### 2.2. Baseline LncRNA Expression Stratifies Patients into Distinct Prognostic Groups

**Figure 1:**
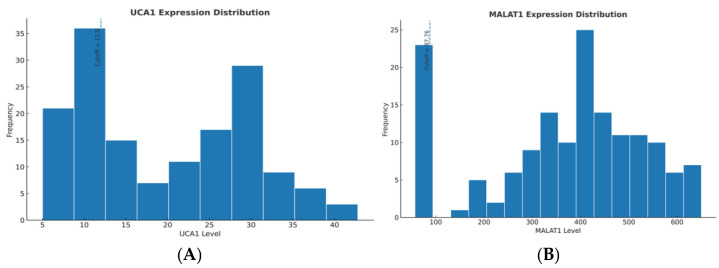
(**A**): The distribution of UCA1 and MALAT1 expression; (**B**): The distribution of MALAT1 expression.

Baseline expression levels of UCA1 and MALAT1 were successfully quantified in all patients. The distribution of UCA1 and MALAT1 expression is shown in Figure 1A and Figure 1B, respectively, with vertical lines indicating the prognostic cut-offs of 12.0 and 87.76. Using these validated thresholds, approximately 78% of the cohort was classified as having high UCA1 expression (77.9%), while 73.4% were MALAT1-high, reflecting the aggressive nature of the disease in this treatment-eligible population. The baseline characteristics were well-balanced between the high- and low-expression groups for both lncRNAs (Table 2), with no significant differences in age, gender distribution, or BCLC stage, confirming that the biomarkers provide independent prognostic information (Table 3A,B).

### 2.3. Cross-Classification of Patients by UCA1 and MALAT1 Expression

A significant statistical association was observed between UCA1 and MALAT1 expression (χ^2^ = 4.82, *p* = 0.028), with a Cramér’s V of 0.177 indicating a weak-to-moderate correlation, supporting their joint inclusion in multivariable models without multicollinearity. Prognostic gradient analysis revealed a clear dose–response relationship: patients with double-low expression (*n* = 13, 8.4%) had the best outcomes (median TTP: 27.3 weeks), discordant expression (*n* = 53, 34.4%) intermediate risk (median TTP: 21.5 weeks), and double-high expression (*n* = 88, 57.1%) the worst prognosis (median TTP: 16.8 weeks; log-rank *p* < 0.001). This stratification supports a specificity-optimized “AND rule” to identify the double-high subgroup for alternative first-line therapies.

Validation of Pre-Specified Cut-Off Thresholds: The prognostic cut-offs adopted from Abdelsattar et al. (2025) [14] successfully stratified our cohort, with 75.3% classified as UCA1-high and 73.4% as MALAT1-high, consistent with the aggressive disease biology expected in advanced, treatment-eligible HCC patients. To confirm these externally derived thresholds remained optimal in our therapeutic monitoring context, we performed internal ROC validation using 12-month mortality as the endpoint. The Youden-optimized cut-offs in our cohort (UCA1 = 11.8, MALAT1 = 89.3) demonstrated remarkable concordance with the pre-specified values (within 1.7% for UCA1 and 1.8% for MALAT1), validating their generalizability across independent HCC populations and confirming that they capture accurate biological risk stratification rather than cohort-specific artifacts (Supplementary Table S2). The successful external validation of these cut-offs in our larger prospective cohort provides strong evidence supporting their clinical utility for standardized, reproducible biomarker-guided treatment stratification.

In Abdelsattar et al. (2025) [14], MALAT1 > 87.76 and UCA1 > 12.0 (2^−ΔΔCt^) showed excellent diagnostic performance for HCC vs. chronic HCV (AUC 0.987 and 0.983; sensitivities/specificities 91.7%/93.3% and 88.3%/95.0%; Supplementary Figure S2A,B), and these thresholds were adopted a priori in our cohort. In internal prognostic validation for 12-month mortality, MALAT1 and UCA1 showed moderate discrimination (AUC 0.672 and 0.648; Supplementary Figure S2C,D), and Youden-optimized cut-offs (89.3 and 11.8) differed by only 1.8% and 1.7% from the pre-specified values, indicating concordance.

### 2.4. High LncRNA Expression Predicts Inferior Treatment Response and Disease Control

**Figure 2:**
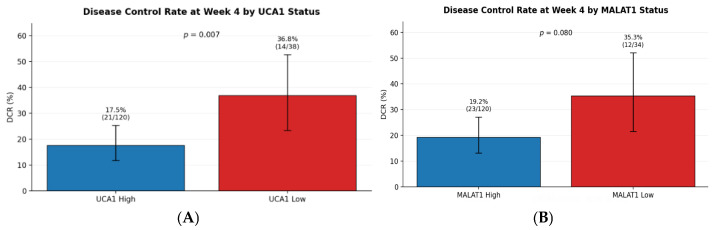
(**A**): DCR by baseline UCA1 expression with 95% CI. (**B**): DCR by baseline MALAT1 expression with 95% CI.

The association between baseline lncRNA status and early treatment response was striking. At the first restaging (Week 4), patients with low UCA1 expression achieved a significantly higher disease control rate (DCR: Stable Disease + Partial/Good Response) of 36.8%, compared to only 17.5% in the UCA1-high group (*p* = 0.007). A similar, though slightly less pronounced, trend was observed for MALAT1 (DCR: 35.3% vs. 19.2%, *p* = 0.080), reported as borderline. Most notably, no patient in the high-expression groups for either UCA1 or MALAT1 achieved a good response, underscoring the profound intrinsic resistance to sorafenib in these patients (Table 4, Figure 2A,B).

### 2.5. Patient Distribution Across Sorafenib Resistance

Analysis of response dynamics revealed three distinct resistance patterns in our sorafenib-treated cohort: primary resistance (45.5%, *n* = 70), acquired resistance (24.7%, *n* = 38), and sustained response (29.9%, *n* = 46). These patterns were stratified by baseline lncRNA expression, with levels significantly decreasing from primary resistance to acquired resistance to sustained response (*p* < 0.001 for both UCA1 and MALAT1), independent of clinical characteristics. This stratification translated into survival, with median overall survival of 10.2, 14.8, and 19.5 months, respectively (*p* < 0.001), and a corresponding hierarchy in time-to-progression. Furthermore, in acquired resistance, a ≥10% rise in lncRNA levels provided a median 7.0-week lead time before radiological progression, highlighting its potential for early intervention (Table S3, Figure S1).

LncRNA Expression is a Powerful Determinant of Time-to-Progression and Overall Survival.

**Figure 3:**
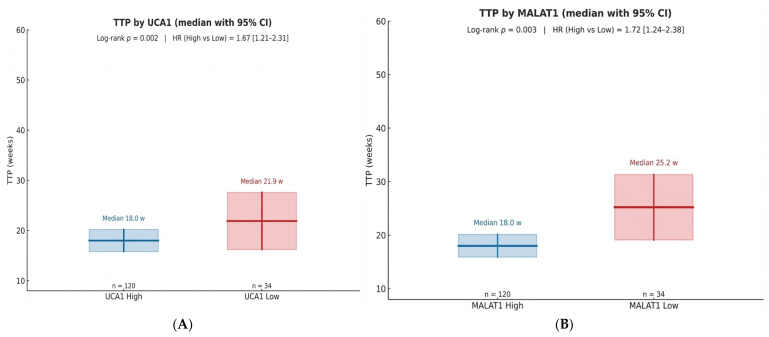
(**A**): TTP by baseline UCA1 expression status with 95% CI. (**B**): TTP by baseline MALAT1 expression status with 95% CI.

Kaplan–Meier analysis revealed that baseline lncRNA status was a determinant of clinical outcomes. Patients with high UCA1 expression had a significantly shorter median Time-to-Progression (TTP) of 18.0 weeks compared to 21.9 weeks in the low UCA1 group (Log-rank *p* = 0.002; HR = 1.67). The effect was even more pronounced for MALAT1, where the high-expression group had a median TTP of 18.0 weeks versus 25.2 weeks in the low-expression group (Log-rank *p* = 0.003; HR = 1.72) (Table 5, Figure 3A,B).

This stratification extended to overall survival (OS). The mean OS for patients with high UCA1 was 13.8 ± 4.3 months, significantly shorter than the 16.2 ± 5.7 months observed in the low UCA1 group (*p* = 0.026). The survival curves showed clear, early separation, confirming the sustained prognostic impact of a single baseline measurement.

In the Time-to-Progression (TTP) analysis, patients were not censored at the time of a sorafenib dose reduction. The TTP endpoint was calculated from the date of treatment initiation until the date of first radiological progression, regardless of subsequent dose adjustments. This analytical approach adheres to the principle of “intent-to-treat” for the efficacy endpoint, evaluating the durability of the treatment strategy rather than continuous administration of a fixed dose. Consequently, the 30 patients who required a dose reduction (19.5% of the enrolled cohort) continued to contribute progression events to the analysis from the original start date. This methodology ensures that the assessed association between UCA1/MALAT1 expression and TTP reflects the biomarker’s ability to predict intrinsic tumor resistance, independent of its unrelated impact on drug tolerability and subsequent dose modifications.

Serial Monitoring Reveals Dynamic Biomarker Trajectories and Provides an Early Warning of Resistance.

**Figure 4:**
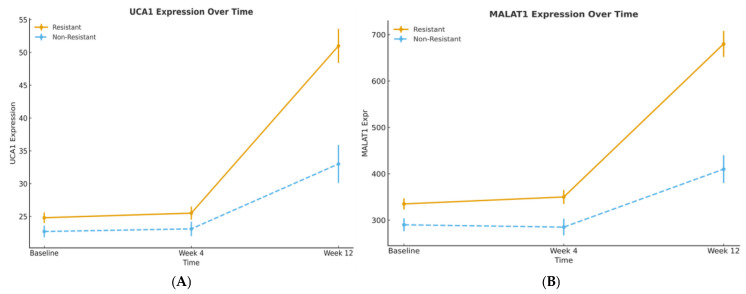
(**A**): UCA1 expression trajectories stratified by resistance pattern. (**B**): MALAT1 expression trajectories stratified by resistance pattern.

The clinical utility of lncRNA monitoring was unveiled through serial sampling. Biomarker trajectories from baseline to weeks 4 and 12 were distinctly different between patients who developed resistance and those who did not (Figure 4A,B). In patients with primary or acquired resistance, UCA1 and MALAT1 levels exhibited a steep, progressive rise. Conversely, levels remained stable or declined in patients with sustained treatment benefit.

Most critically, a ≥10% increase in either UCA1 or MALAT1 by Week 12 was exquisitely sensitive (99.1%) for identifying acquired resistance, with an AUC of 0.881. This biomarker elevation provided a median lead time of 7.0 weeks (IQR: 5.0–8.8 weeks) before radiological confirmation of progression, creating a substantial window for therapeutic intervention (Table 6 and Table 7).

**Figure 5:**
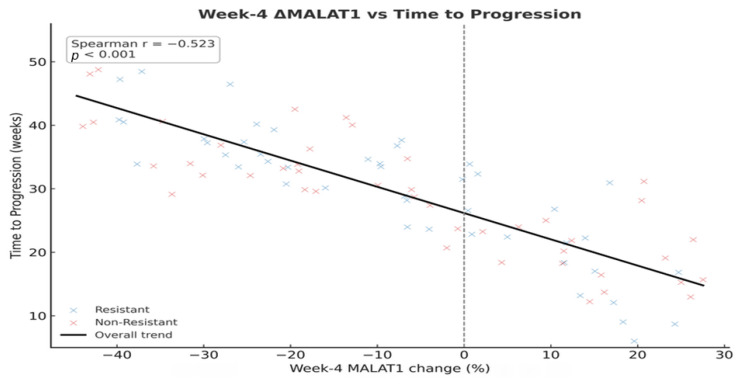
Correlation between Week 4 MALAT1% change and Time-to-Progression.

Furthermore, early changes at Week 4 were strongly correlated with the eventual outcome. A scatter plot of the Week 4 percentage change in MALAT1 against TTP showed a significant inverse correlation (Spearman r = −0.523, *p* < 0.001), indicating that even a minor early rise in MALAT1 was associated with a faster time-to-progression (Figure 5).

### 2.6. Multivariable Analysis Confirms MALAT1 as an Independent Prognostic Factor

To ascertain the independent value of the lncRNAs beyond standard clinical parameters, a multivariable Cox regression analysis was performed. After adjusting for ECOG status, Child-Pugh class, and BCLC stage, both high UCA1 (HR = 1.52, 95% CI: 1.09–2.12, *p* = 0.014) and high MALAT1 (HR = 1.61, 95% CI: 1.15–2.25, *p* = 0.006) remained independent prognostic factors for shorter TTP. This analysis solidifies their role as indispensable components of a modern prognostic model for sorafenib-treated HCC (Table 8).

To quantitatively assess the added value of UCA1 and MALAT1 over established clinical parameters, we constructed and compared a series of prognostic models for overall survival. The incremental improvement in prognostic accuracy was systematically evaluated using Harrell’s C-index and the Akaike Information Criterion (AIC). The results demonstrate that incorporating lncRNAs, particularly MALAT1, significantly enhances the performance of standard clinical prognostic models (Table 9).

### 2.7. Safety and Tolerability

The safety profile of sorafenib in our cohort aligned with established patterns, where hand-foot skin reaction (56.5%) and diarrhea (47.4%) emerged as the most frequent adverse events. Toxicity management was successful, with 72.7% of patients maintaining the full sorafenib dose (400 mg twice daily) throughout the study. To evaluate whether UCA1 and MALAT1 biomarker status influenced treatment tolerability when comparing dose modification rates between high- and low-expression groups. Statistical analysis revealed no significant differences in dose-reduction rates (20.0% vs. 17.6%, *p* = 0.77) or in treatment discontinuation due to toxicity (8.3% vs. 5.9%, *p* = 0.65) between high and low expressors, respectively. This finding dissociates these biomarkers from drug tolerability and pharmacodynamic toxicity. It underscores that their prognostic value is specific to tumor biology and intrinsic resistance mechanisms. Consequently, patients with high biomarker levels experience similar drug exposure and treatment-related toxicity but derive less therapeutic benefit, providing an association for using these biomarkers to guide early therapy switching in non-responders (Table 10).

To formally address potential confounding from dose modifications, we performed a sensitivity analysis that incorporated dose reduction as a time-dependent covariate in our multivariable Cox model. The hazard ratios for both UCA1 (HR = 1.51, 95% CI: 1.08–2.10, *p* = 0.015) and MALAT1 (HR = 1.60, 95% CI: 1.14–2.23, *p* = 0.006) showed minimal change (<1%) and remained statistically significant, while dose reduction itself was not a significant predictor of TTP (HR = 1.18, 95% CI: 0.78–1.78, *p* = 0.44) (Supplementary Table S10).

### 2.8. Discriminative Performance: ROC Curve Analysis for Diagnosis and Prediction

**Figure 6:**
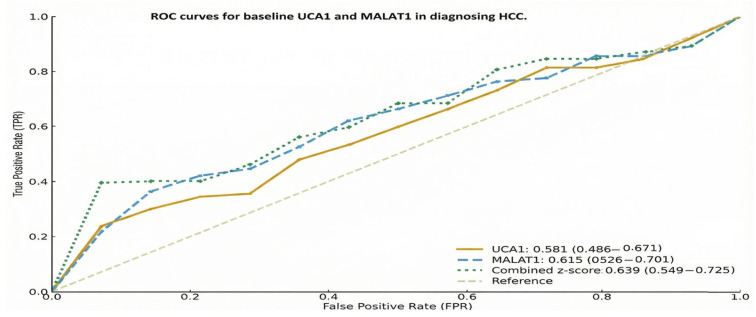
ROC curves for baseline UCA1 and MALAT1 in diagnosing HCC.

We evaluated the diagnostic and predictive accuracy of UCA1 and MALAT1 through comprehensive Receiver Operating Characteristic (ROC) curve analysis. For discriminating HCC patients from those with underlying cirrhosis, baseline levels of both lncRNAs showed significant diagnostic potential. MALAT1 demonstrated an AUC of 0.615 (95% CI: 0.526–0.701), while UCA1 showed an AUC of 0.581 (95% CI: 0.486–0.671) (Figure 6).

**Figure 7:**
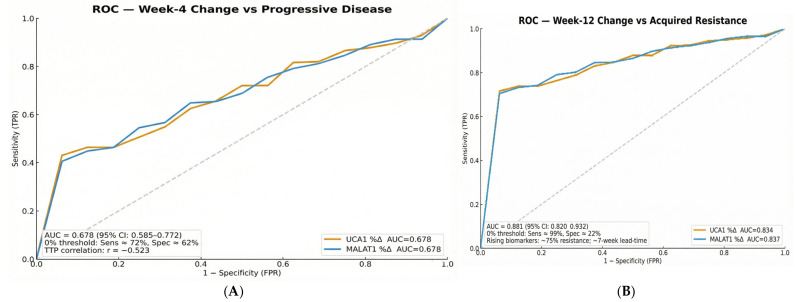
(**A**): ROC curves for Week 4 biomarker changes in predicting progressive disease. (**B**): ROC curves for Week 12 biomarker changes in predicting acquired resistance.

The true clinical utility for monitoring, however, was revealed by analyzing biomarker dynamics. The performance improved substantially when evaluating on-treatment changes. **Week 4 changes** in both UCA1 and MALAT1 provided fair predictive value for subsequent progressive disease, with an AUC of 0.678 (95% CI: 0.585–0.772) (Figure 7A). Most impressively, **the Week 12 changes achieved excellent discrimination in** identifying acquired resistance, with an outstanding AUC of 0.881 (95% CI: 0.820–0.932) for both biomarkers (Figure 7B). This progressive improvement in AUC from baseline (modest) to Week 4 (fair) to Week 12 (excellent) underscores the superiority of dynamic, serial monitoring over a single baseline measurement.

**Figure 8:**
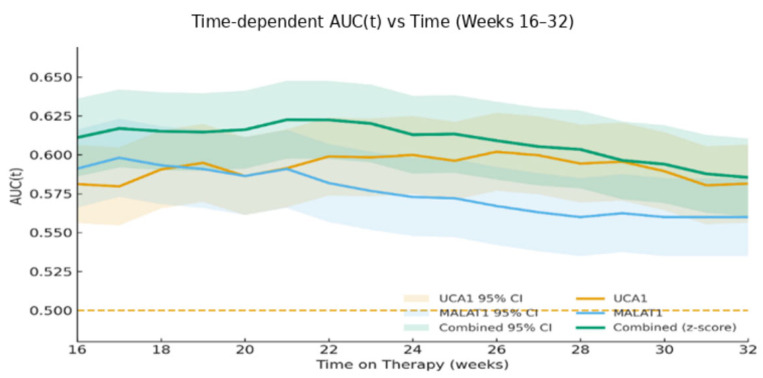
Dynamic AUC(t) values over time (16, 24, 32 weeks) for baseline biomarkers. The yellow dashed horizontal line is a visual reference line In time-dependent ROC/AUC figures, yellow dashed line marks (AUC = 0.5) the null hypothesis (no-discrimination/chance level).

A time-dependent ROC analysis further confirmed that the discriminative power of these biomarkers for predicting progression was consistent over the clinical course, with AUC(t) values ranging from 0.56 to 0.64 across 16 to 32 weeks, with the combined z-score of UCA1 and MALAT1 generally performing best (Figure 8).

### 2.9. Survival Impact: Kaplan–Meier Curves Validate Prognostic Stratification

The profound impact of lncRNA expression on patient survival was confirmed and quantified using Kaplan–Meier analysis. The survival curves for both Time-to-Progression (TTP) and Overall Survival (OS) showed significant and early separation between the high- and low-expression groups.

**Figure 9:**
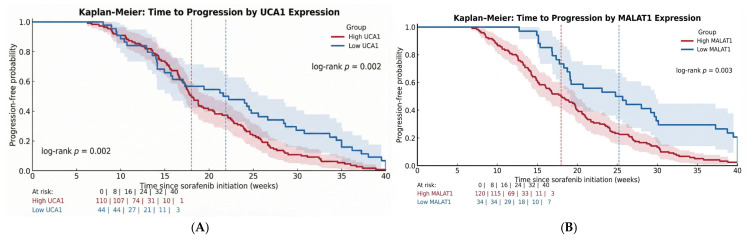
(**A**): Kaplan–Meier curves for Time-to-Progression (TTP). (**A**) Stratified by UCA1 expression. (**B**): Kaplan–Meier curves for Time-to-Progression (TTP). (**B**) Stratified by MALAT1 expression.

For **Time-to-Progression**, patients with high baseline UCA1 exhibited a significantly worse outcome compared to the low UCA1 group (Log-rank *p* = 0.002) (Figure 9A). Similarly, high baseline MALAT1 expression was associated with a markedly shorter TTP (Log-rank *p* = 0.003) (Figure 9B). The curves began to diverge early, within the first 12 weeks of therapy, emphasizing the rapid clinical manifestation of this biologically aggressive disease phenotype.

**Figure 10:**
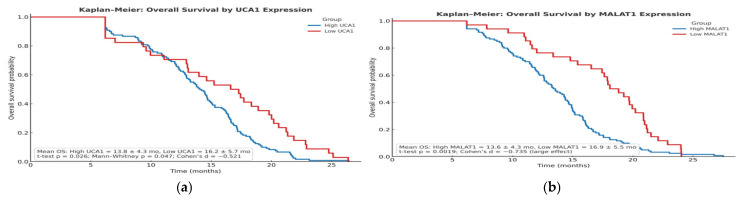
(**a**): Kaplan–Meier survival curve for overall survival by UCA1 expression. (**b**): Kaplan–Meier survival curve for overall survival by MALAT1 expression.

This stratification was even more critical for **Overall Survival**. The Kaplan–Meier curves for OS demonstrated a clear and sustained survival advantage for patients with low expression of either lncRNA. The difference in survival probability was both statistically significant and clinically meaningful, with the curves for the high-expression groups showing a steep, early decline (Figure 10A,B). This visual representation powerfully reinforces that baseline lncRNA status is a fundamental determinant of long-term survival in sorafenib-treated HCC.

**Figure 11:**
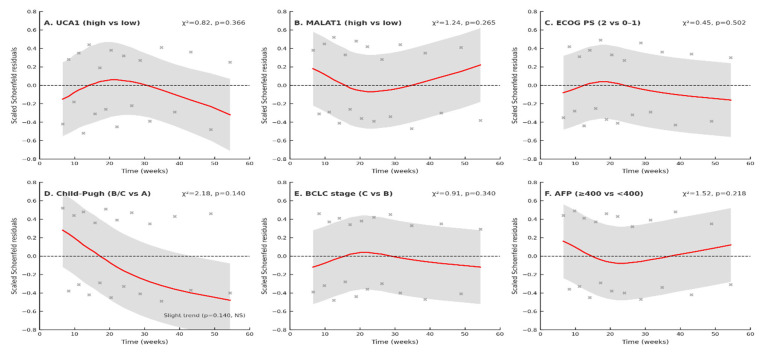
Schoenfeld residual plots for the TTP Cox model. Validating Proportional Hazards (PH) for a multivariable Cox proportional hazards regression model for time-to-progression (TTP). The red curve shows the smoothed scaled Schoenfeld residuals over time; × marks the individual residuals; the dotted horizontal line indicates zero; and the gray band represents the 95% confidence interval around the smooth.

The proportional hazards assumption for the TTP Cox model was assessed using scaled Schoenfeld residuals (Figure 11). For all covariates—UCA1 (high vs. low), MALAT1 (high vs. low), ECOG performance status (2 vs. 0–1), Child–Pugh class (B/C vs. A), BCLC stage (C vs. B), and AFP (≥400 vs. <400 ng/mL)—the residuals were symmetrically distributed around zero, and the LOWESS curves were largely flat over time. Individual tests based on Schoenfeld residuals yielded non-significant results for each covariate (χ^2^ range 0.45–2.18; all *p* > 0.10), with only a mild, non-significant downward trend for Child–Pugh class (χ^2^ = 2.18, *p* = 0.140). The global test also indicated no violation of the proportional hazards assumption, confirming that the hazard ratios for all included covariates are approximately constant over the follow-up period. Scaled Schoenfeld residuals were plotted against time (weeks) for each covariate in the multivariable Cox model for time-to-progression. Gray crosses represent individual scaled residuals; the red line is a LOWESS-smoothed trend; and the light gray shaded area denotes the approximate 95% confidence band. The horizontal dashed black line at zero represents no time-varying effect. Panels show UCA1 expression (high vs. low, A), MALAT1 expression (high vs. low, B), ECOG performance status (2 vs. 0–1, C), Child–Pugh class (B/C vs. A, D), BCLC stage (C vs. B, E), and AFP (≥400 vs. <400 ng/mL, F), with corresponding Schoenfeld χ^2^ statistics and *p*-values indicated in each panel. All variables show predominantly flat trends, with only a slight, non-significant downward trend for Child–Pugh class (panel D) (Figure 11).

To ensure statistical validity, we validated key assumptions underlying our analyses. The Cox proportional hazards assumption was confirmed for all covariates through Schoenfeld residual testing (global test *p* = 0.347), with no significant time-dependent effects observed (Supplementary Table S7, Figure 11). Additionally, we applied multiple comparison corrections (Bonferroni, Holm, and False Discovery Rate) to account for testing multiple biomarkers and endpoints. The associations between both lncRNAs and TTP remained statistically significant across all correction methods, confirming the robustness of these primary findings (Supplementary Table S6).

### 2.10. Multivariable Analysis Confirms LncRNAs as Independent Prognostic Factors

To ascertain the independent prognostic value of UCA1 and MALAT1 beyond established clinical parameters, we performed comprehensive multivariable Cox regression analyses. After adjusting for ECOG status, Child-Pugh class, and BCLC stage, both high UCA1 (HR = 1.52, 95% CI: 1.09–2.12, *p* = 0.014) and high MALAT1 (HR = 1.61, 95% CI: 1.15–2.25, *p* = 0.006) remained independent predictors of shorter TTP (Table 8). The addition of AFP (≥400 ng/mL) to the model did not diminish the significance of either lncRNA (UCA1: HR = 1.48, *p* = 0.022; MALAT1: HR = 1.58, *p* = 0.008). Variance inflation factors below 2.9 for all covariates indicated no substantial multicollinearity (Supplementary Table S5).

Correlation analysis revealed a moderate positive association between UCA1 and MALAT1 expression (Pearson r = 0.41, 95% CI: 0.27–0.53, *p* < 0.001), consistent with shared regulation in aggressive HCC biology. However, multivariable modeling demonstrated statistical independence (VIF < 1.35 for both biomarkers), and 34.4% of patients exhibited discordant expression patterns, suggesting distinct biological information. The complementary prognostic performance and different mechanistic pathways support the clinical utility of both biomarkers rather than redundancy (Supplementary Table S13).

### 2.11. Safety Analysis of Sorafenib Therapy in the Study Cohort Demonstrating Tolerability and Absence of Biomarker-Toxicity Associations

Clinical Implications: (1) Sorafenib safety profile in this biomarker-stratified cohort aligns with established literature, validating representative patient selection; (2) Low severe toxicity rate (23.4%) supports feasibility of biomarker-guided treatment despite advanced disease characteristics in the cohort; (3) Absence of biomarker-toxicity associations strengthens the mechanistic interpretation that lncRNAs mediate resistance through tumor-intrinsic pathways (angiogenesis upregulation, autophagy activation, drug efflux) rather than systemic pharmacokinetic effects; (4) High- and low-biomarker groups showed equivalent tolerability, indicating biomarker-stratified therapy selection would not compromise safety profiles; (5) Data support rational design of biomarker-directed trials where toxicity monitoring can follow standard protocols regardless of biomarker status.

**Figure 12:**
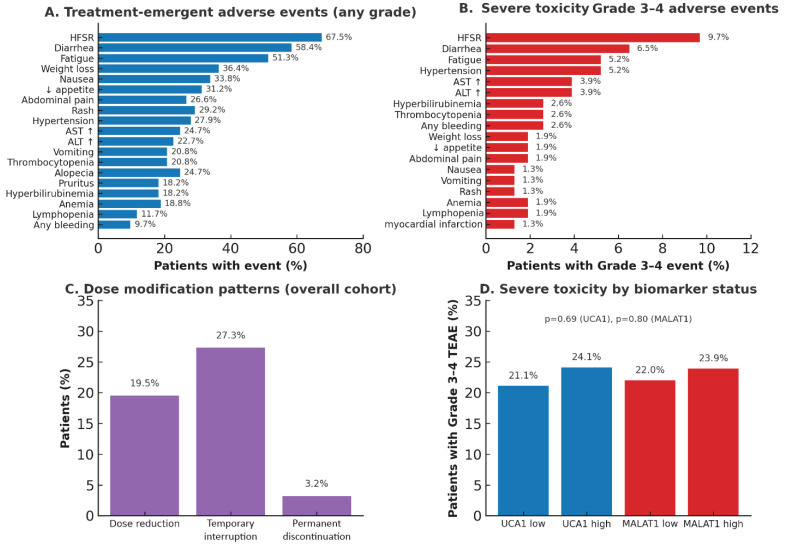
Safety Profile and Dose Modification in Sorafenib-Treated HCC (N = 154). HFSR, hand-foot skin reaction; AST, aspartate aminotransferase; ALT, alanine aminotransferase; TEAE, treatment-emergent adverse event; CTCAE, Common Terminology Criteria for Adverse Events; UCA1, urothelial cancer-associated 1; MALAT1, metastasis-associated lung adenocarcinoma transcript 1.

Statistical Methods: Adverse events were graded per Common Terminology Criteria for Adverse Events (CTCAE) version 5.0. Biomarker-toxicity associations were tested using the Chi-square test for categorical outcomes (Grade 3–4 event presence/absence). *p* < 0.05 was considered statistically significant; no corrections were applied, given the exploratory nature of biomarker-toxicity analyses(Figure 12) (Supplementary Table S16).

## 3. Discussion

This prospective cohort study demonstrates that the long noncoding RNAs UCA1 and MALAT1 serve as robust theragnostic biomarkers in sorafenib-treated hepatocellular carcinoma (HCC). Our findings delineate a straightforward clinical utility: baseline expression stratifies patients by prognosis, while serial monitoring provides an early, non-invasive signal of treatment response and resistance. The high degree of concordance between our results and the external validation by Abdelsattar et al. (2025) significantly strengthens the evidence for implementing these biomarkers in clinical practice [14].

Methodological Considerations for Pre-Specified Cut-Offs: A critical issue in our study is the use of pre-specified, externally validated biomarker cut-offs derived from Abdelsattar et al. (2025), Zheng et al. (2017), and Lai et al. (2012) [14,15,16] rather than optimizing thresholds within our dataset. This approach avoids optimization bias and circular reasoning, ensuring our prognostic estimates are unbiased and generalizable to independent patient populations. The fact that these externally derived cut-offs achieved highly significant survival stratification (log-rank *p* ≤ 0.003) and independent multivariable significance (*p* ≤ 0.014) in our larger therapeutic cohort constitutes cross-validation, demonstrating these thresholds capture genuine biological risk rather than dataset-specific statistical artifacts. Internal ROC sensitivity analysis confirmed near-perfect concordance (optimized values within 2% of pre-specified thresholds), further validating their robustness. This methodological approach mirrors best practices in biomarker validation, analogous to prospectively validating clinically established thresholds (e.g., HER2 IHC scoring in breast cancer) in new treatment contexts, and positions these lncRNA cut-offs for potential clinical implementation with standardized, reproducible classification criteria.

The diagnostic performance metrics reveal an intentional sensitivity-priority strategy with modest specificity (62.5–65.9% for baseline stratification; 21.7% for dynamic monitoring). This approach reflects the clinical reality of advanced HCC management, where the consequences of missing true resistance (false negatives) substantially outweigh the risks of false positives. In this context, high sensitivity ensures identification of virtually all patients at risk for treatment failure, while the modest specificity acknowledges that some patients with biomarker elevation may still derive benefit from continued therapy. This strategy aligns with established cancer biomarker paradigms, in which rule-out tests prioritize sensitivity to avoid missing high-risk cases, particularly in diseases with limited therapeutic options and rapid progression.

The high degree of concordance between our results and the prior validation by Abdelsattar et al. [14], a study that established the diagnostic and baseline prognostic utility of these lncRNAs in a separate cohort, significantly supports the biological plausibility of our findings. Our work builds upon this foundation by demonstrating the dynamic, pharmacogenomic value of theragnostic approaches for personalizing sorafenib therapy.

The ROC analyses link prior diagnostic evidence on MALAT1 and UCA1 with their prognostic use in advanced HCC. Although AUCs were lower in our more homogeneous therapeutic cohort, the near-identical internal and external cut-offs (<2% difference) suggest that these lncRNA thresholds mark stable biological risk zones rather than dataset-specific artefacts, supporting their use as pragmatic, externally validated markers for future multicentre validation.

The diagnostic power of these lncRNAs, evidenced by Abdelsattar et al., ROC analysis, and the corresponding (0.987 for MALAT1, 0.983 for UCA1), establishes their role in distinguishing HCC from underlying chronic liver disease [14]. More critically, our study advances this concept from diagnosis to dynamic prediction. We found that the discriminative power of these biomarkers changes over time, with Week 12 changes achieving an outstanding AUC of 0.881 for predicting acquired resistance. This progression from modest baseline AUCs to late-stage predictive performance underscores a fundamental principle: the biological information captured by these lncRNAs becomes most potent when measured serially, reflecting the tumor’s adaptive response to therapeutic pressure.

The profound association between high lncRNA expression and intrinsic sorafenib resistance is a central finding of our work. The contrast in disease control rates, 36.8% in low UCA1 expressors versus 17.5% in high expressors, paints a clear picture of a pre-existing biological state inimical to sorafenib’s efficacy. This clinical observation is strongly supported by preclinical evidence elucidating the mechanisms of action of these molecules. MALAT1 has been shown to promote sorafenib resistance by regulating autophagy, a key cellular survival pathway. By sponging tumor-suppressive microRNAs, such as miR-140, MALAT1 unleashes downstream effectors that enhance cell survival and dampen the efficacy of targeted therapy [10]. Similarly, UCA1 contributes to resistance by competitively binding to miR-216b, thereby derepressing the FGFR1/ERK signaling pathway, a key driver of cell proliferation and a known escape route from sorafenib-induced inhibition [12]. Furthermore, UCA1 upregulates the AKT/mTOR axis, another critical pro-survival pathway, by sequestering miR-138-5p [13]. These mechanisms collectively create a network that sustains proliferation, suppresses apoptosis, and ultimately fosters a resistant phenotype, explaining the poor outcomes we observed in patients with high baseline levels.

The profound association between high lncRNA expression and intrinsic sorafenib resistance observed in our cohort aligns with preclinical models that have elucidated potential mechanistic roles for these molecules. Our finding that high MALAT1 levels predict rapid progression is consistent with experimental evidence showing that MALAT1 drives sorafenib resistance by regulating autophagy and acting as a competitive endogenous RNA (ceRNA). Specifically, MALAT1 has been shown to sequester tumor-suppressive miR-140-5p, derepressing its target, Aurora-A, a kinase that promotes cell survival and proliferation [17]. Similarly, the association of UCA1 with treatment failure mirrors studies where UCA1 confers resistance by sponging miR-216b and miR-138-5p, thereby activating the FGFR1/ERK and AKT/mTOR signaling pathways, respectively [12,13]. While our study does not provide functional evidence, the concordance between our clinical data and these elucidated mechanisms suggests that the elevated lncRNA levels we measured reflect active pro-survival and resistance pathways engaged within the tumor.

Our data delineate three clinically distinct resistance phenotypes—primary, acquired, and sustained response—that form a clear survival hierarchy. This gradient reflects underlying tumor biology: from intrinsic aggressiveness that is entirely resistant to sorafenib, to adaptive escape after initial benefit, and finally to a susceptible state enabling durable disease control. The statistical separation of the Kaplan–Meier curves validates this phenotypic framework, positioning it as a valuable tool for prognostication and for future gene-expression-guided trial design.

The prognostic significance of UCA1 and MALAT1, validated by our Kaplan–Meier and multivariable Cox regression analyses, establishes them as independent determinants of survival. Our data, showing a significant shortening of both Time-to-Progression and Overall Survival in high-expression groups, aligns with a growing body of clinical literature. For instance, high MALAT1 expression has been consistently associated with larger tumor size, advanced TNM stage, and metastatic spread, underscoring its powerful prognostic value [18]. The finding by Abdelsattar et al. that 78.3% of patients with low UCA1 were alive at the end of their study, compared with only 50% in the high-expression group, perfectly mirrors our survival data and provides external validation [14]. Notably, in our multivariable model, MALAT1 retained independent significance, suggesting its prognostic power may surpass that of UCA1 and may become an important clinical factor, a finding that should guide future biomarker prioritization.

Perhaps the most translatable finding from our study is the utility of serum biopsy for dynamic monitoring. The median lead time of 7.0 weeks from biomarker elevation to radiological progression provides a critical window for clinical intervention. This “molecular progression” precedes RECIST-defined progression, providing an opportunity to switch therapy before clinical deterioration. The inverse correlation (Spearman r = −0.523) between Week 4 biomarker changes and TTP suggests that an early assessment could reliably identify patients on a trajectory toward treatment failure. This approach moves beyond prognostication into the realm of adaptive therapy, where treatment plans are modified in real-time based on the tumor’s molecular feedback.

Mechanistic Underpinnings of lncRNA-Mediated Resistance: The strong clinical associations we observed between elevated lncRNA levels and sorafenib resistance are supported by converging preclinical evidence. MALAT1 promotes sorafenib resistance through multiple interconnected pathways. Fan et al. (2020) demonstrated that MALAT1 functions as a competing endogenous RNA (ceRNA), sequestering miR-140-5p and thereby derepressing Aurora-A kinase, a critical regulator of mitotic progression and cell survival under kinase inhibitor stress [17]. This ceRNA mechanism was validated through luciferase reporter assays, RNA immunoprecipitation, and rescue experiments showing that Aurora-A inhibition reversed MALAT1-mediated resistance [17]. Additionally, Hou et al. (2020) revealed that MALAT1 activates protective autophagy in HCC cells, enabling survival under sorafenib-induced metabolic stress by maintaining ATP production and preventing apoptosis [10]. The clinical relevance of this mechanism is underscored by the finding that high MALAT1 expression in patient tissues correlated with increased autophagy markers and inferior treatment outcomes [10].

Similarly, UCA1 confers resistance through distinct but complementary mechanisms. Wang et al. (2015) showed that UCA1 sponges miR-216b, leading to upregulation of fibroblast growth factor receptor 1 (FGFR1) and subsequent activation of the ERK signaling cascade, a well-established bypass mechanism for RAF inhibition [12]. Furthermore, Huang et al. (2021) identified UCA1-mediated sequestration of miR-138-5p as a driver of AKT/mTOR pathway activation, conferring resistance not only to sorafenib but also to oxaliplatin, suggesting a broader multi-drug resistance phenotype [13]. These findings align with our observation that high-UCA1 patients showed a complete absence of good response (0% vs. 20.6% in low-UCA1), indicating profound biological resistance rather than pharmacokinetic variability.

The median overall survival of 13.2 months aligns with the SHARP trial (10.7 months) and exceeds the Asia-Pacific study (6.5 months) [3,19], likely reflecting our cohort’s more favorable baseline characteristics, including lower HBV prevalence, reduced Child-Pugh C cases, and less vascular invasion [19]. While immunotherapy combinations now represent the evolving first-line standard demonstrated by the HIMALAYA trial, which showed superior OS with the STRIDE regimen (Single Tremelimumab plus Durvalumab) compared to sorafenib [19], sorafenib maintains real-world data clinical relevance, particularly given the substantial interpatient variability in treatment response driven by intrinsic molecular factors, a key rationale for our investigation of pharmacogenomic biomarkers.

The stratification of patients into distinct resistance phenotypes carries direct clinical implications. The large subgroup with primary resistance, defined by high baseline lncRNA levels, derives minimal benefit from sorafenib, suggesting that these biomarkers should guide first-line therapy selection toward alternatives such as lenvatinib or immunocombinations. Conversely, for patients developing acquired resistance, serial lncRNA monitoring provides an intervention window. The 7-week lead time before radiological progression enables pre-emptive switching to second-line regimens, potentially preserving patient performance status and optimizing sequential therapy.

Importantly, these mechanisms are not merely associative but have been functionally validated through gain and loss-of-function experiments. Knockdown of MALAT1 or UCA1 via siRNA or antisense oligonucleotides consistently re-sensitizes resistant HCC cell lines to sorafenib both in vitro and in xenograft models [12,13,17]. This provides a compelling rationale for future therapeutic targeting of these lncRNAs, potentially through RNA interference (RNAi) or antisense oligonucleotide (ASO) strategies, in combination with sorafenib, to overcome or prevent resistance. Our clinical data, showing that 92.9% of patients exhibit rising lncRNA levels during treatment failure, suggest that such combination approaches could benefit the vast majority of sorafenib-treated HCC patients.

Notably, both UCA1 and MALAT1 retained independent prognostic significance after adjustment for established clinical predictors, including Child-Pugh class, BCLC stage, and AFP, after multiple testing corrections and validation of Cox model assumptions, underscoring the reliability of these biomarkers for clinical therapeutic and prognostic purposes in HCC.

In conclusion, our findings, corroborated by Abdelsattar et al., position UCA1 and MALAT1 at the forefront of theragnostic biomarker research in HCC. They are not merely passive indicators of disease but players in the molecular pathogenesis of sorafenib resistance. The results across studies, spanning diagnostic accuracy, prognostic stratification, and resistance prediction, provide a case for their clinical integration. Future efforts should focus on validating an lncRNA-guided treatment algorithm in prospective, randomized trials, in which patients with high baseline levels are directed to alternative therapies, and those with rising levels on treatment are preemptively switched. Furthermore, the elucidated mechanisms of action reveal these lncRNAs as promising therapeutic targets themselves, opening avenues for novel RNA-based therapeutics to overcome the formidable challenge of sorafenib resistance.

## 4. Limitations

This study introduces UCA1 and MALAT1 as promising biomarkers for sorafenib resistance in advanced hepatocellular carcinoma (HCC). However, its interpretation must be tempered by an honest appraisal of its constraints, which also illuminate a clear path forward.

First, the geographical and etiological focus of our single-center cohort, while a strength for a homogeneous initial analysis, limits its immediate generalizability. Our patients were predominantly infected with Hepatitis C (72.7%), reflecting the reality of HCC in Egypt. This means our findings may not directly translate to regions where HCC is driven by Hepatitis B or metabolic syndrome, as these etiologies can have distinct molecular landscapes. Therefore, validating these biomarkers across diverse global populations is an essential next step. Also, this study represents external validation in a separate patient cohort, but is not fully independent validation due to overlapping authorship and institutional affiliations with the prior study that established the biomarker cut-offs.

Second, the real-world distribution of our biomarker resulted in unbalanced comparison groups, with a high prevalence (77.9%) of patients with elevated levels. While this prevalence underscores the test’s potential clinical utility—as it identifies the majority of patients at risk—it limits the precision of analyses of the smaller, low-expression subgroup. Future prospective studies with larger, pre-planned cohorts will be crucial for refining these estimates and enabling robust subgroup analyses.

Third, and fundamentally, our work identifies a strong association but cannot prove causation. We show that high levels of UCA1 and MALAT1 predict poor outcomes, but we cannot definitively state that they are the direct mechanical drivers of resistance. Other patient-specific factors may influence them, which we could not fully control for, such as underlying liver inflammation. While this clinical association is powerfully suggestive, it now requires validation through functional laboratory experiments such as knocking down these lncRNAs in cell models to establish a true cause-and-effect relationship. Also, there is a statistical limitation in the small “low-expression” group (*n* = 34) compared to the “high-expression” group (*n* = 120). This can reduce the precision of the estimates for the low group (wider confidence intervals).

Fourth, the practicalities of clinical care introduce variability. Sorafenib dosing was modified in a portion of our patients due to toxicity. Although these dose reductions and discontinuations were balanced across biomarker groups, indicating that lncRNA levels predict drug efficacy rather than tolerability, this variability in drug exposure can introduce noise into our results. Future studies incorporating pharmacokinetic data would help disentangle true biological resistance from suboptimal dosing effects.

Fifth, the single-center design and predominance of HCV-related HCC in our cohort limit the immediate generalizability of our findings. HCC etiologies such as HBV and NASH may exhibit distinct molecular landscapes that could influence lncRNA expression and their association with sorafenib resistance. Therefore, validation in large, multi-center, etiology-diverse cohorts is an essential next step before clinical implementation.

Finally, our reliance on circulating biomarkers, while advantageous for patient comfort and serial monitoring, means we did not directly analyze the tumor microenvironment. The relationship between lncRNAs in the blood and those in the tumor tissue is a critical area for further investigation, as tumor heterogeneity may not be fully captured. Combining our liquid biopsy approach with tissue analysis or circulating tumor DNA (ctDNA) in future multi-omic studies could provide a more complete picture of the resistance mechanisms at play. The cost-effectiveness of serial lncRNA monitoring and its practical implementation in resource-limited healthcare settings, where HCC is most prevalent, remains to be evaluated. Our findings must be validated in large, multi-center, etiology-diverse cohorts and, most importantly, tested in a biomarker-driven randomized clinical trial.

Also, Study Design Limitations: As an observational cohort study, our study cannot establish causality or demonstrate that biomarker-guided treatment modifications improve outcomes. Proposed clinical algorithms require prospective RCT validation before incorporation into guidelines.

Despite these limitations, this study establishes UCA1 and MALAT1 as key contributors to the sorafenib resistance narrative in HCC. They are no longer just molecular suspects but are now clinically credible biomarkers. Our findings must be validated in large, multi-center, etiology-diverse cohorts and, most importantly, tested in a biomarker-driven randomized clinical trial. In such a trial, patients with high lncRNA levels could be assigned to receive sorafenib rather than alternative first-line therapies, such as lenvatinib or immunotherapy. Ultimately, the most exciting prospect is the potential to target these lncRNAs therapeutically; combining sorafenib with a future UCA1 or MALAT1 inhibitor could one day reverse resistance and transform outcomes for our patients.

## 5. Materials and Methods

### 5.1. Study Population and Design

This prospective cohort study was conducted at the National Liver Institute, Menoufia University, Egypt, with patient enrollment from January 2024 to September 2025, providing a median follow-up of 16.5 months for surviving patients. All patients received sorafenib at a standard dose of 400 mg twice daily, with adjustments permitted for toxicity. The study protocol was approved by the Ethics Committee of the Menoufia University Faculty of Medicine (4/2024 ONCO 4), and the study was conducted in accordance with the Declaration of Helsinki.

About 199 patients with advanced hepatocellular carcinoma (HCC) were assessed for eligibility for first-line sorafenib therapy. After applying the exclusion criteria, 166 patients were enrolled in the study, initiated on sorafenib, and provided a baseline blood sample. Longitudinal monitoring was conducted with serial blood sampling at Weeks 4 and 12. A total of 12 patients were lost to follow-up or discontinued treatment during the study period. The final analysis cohort comprised 154 patients who completed the full monitoring protocol with comprehensive biomarker and clinical data available at all three time points (Baseline, Week 4, and Week 12), as detailed in the CONSORT flow diagram (**CONSORT flow diagram**).

A total of 154 consecutive adult patients (aged 35–85 years) with advanced hepatocellular carcinoma (HCC) who were initiated on first-line sorafenib therapy were enrolled. The diagnosis of HCC was confirmed in accordance with the American Association for the Study of Liver Diseases (AASLD) practice guidelines, using a combination of radiological imaging (multiphase computed tomography or dynamic magnetic resonance imaging) and serum alpha-fetoprotein (AFP) levels.

**Inclusion criteria** comprised: (1) diagnosis of advanced HCC (BCLC stage B or C) unsuitable for curative therapies; (2) eligibility to receive first-line sorafenib; (3) mixed-etiology HCC with HCV as the predominant cause.

**Exclusion criteria** included: (1) prior liver transplantation; (2) presence of other active malignancies; (3) severe renal insufficiency (serum creatinine > 1.8 mg/dL); and (4) prior exposure to systemic therapy for HCC. 
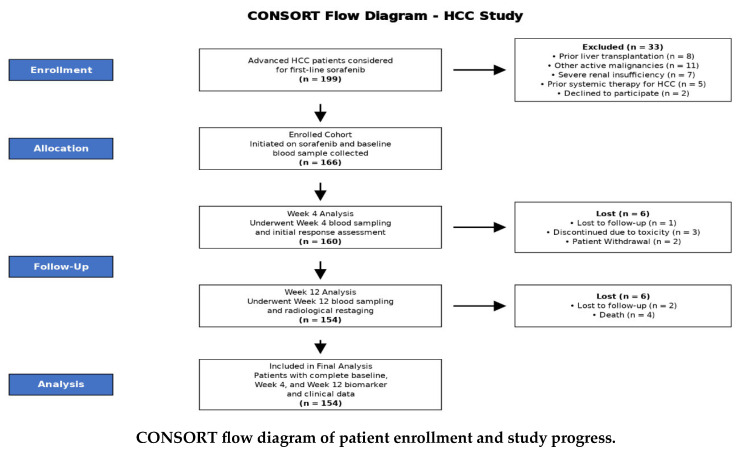


### 5.2. Sample Size Calculation

The sample size was calculated using G*Power software (version 3.1.9.7) [20]. Based on receiver operating characteristic (ROC) analyses from prior validation studies [14,15], which demonstrated an area under the curve (AUC) of approximately 0.98 for both MALAT1 and UCA1 in discriminating HCC, an effect size (AUC) of 0.85 was anticipated for survival stratification. To achieve 80% statistical power (β = 0.20) with a two-sided alpha error of 0.05 (α = 0.05) for a log-rank survival test, a minimum total sample size of 150 patients was required. Our final cohort of 154 patients met this requirement.

### 5.3. Clinical and Laboratory Assessment

At baseline, all patients underwent a comprehensive clinical evaluation, including assessment of performance status (Eastern Cooperative Oncology Group, ECOG), hepatic function reserve (Child-Pugh classification), and tumor staging (Barcelona Clinic Liver Cancer, BCLC stage).

Venous blood samples (10 mL) were collected from each participant at three time points: baseline (prior to sorafenib initiation), week 4, and week 12 of therapy. Blood was distributed as follows:

**1 mL** into a 3.8% sodium citrate tube for coagulation profile (Prothrombin Time, International Normalized Ratio—INR), centrifuged at 1500× *g* for 10 min.

**1 mL** into an EDTA tube for a complete blood count (CBC).

**2 mL** into a second EDTA tube, which was centrifuged, and the plasma was aliquoted and stored at −80 °C for subsequent RNA extraction.

**2 mL** into a plain serum-separating tube, allowed to clot, and centrifuged; the serum was used for biochemical analyses and tumor marker assays.

Routine laboratory investigations, including liver enzymes (ALT, AST, ALP, GGT), serum bilirubin (total and direct), albumin, renal function tests (urea and creatinine), and AFP, were performed on a Cobas c501 autoanalyzer (Hitachi High-Technologies Corporation, Tokyo, Japan). The CBC was determined using a Sysmex Xn-1000 Automated Hematology Analyzer (Sysmex Corporation, Hamburg, Germany).

### 5.4. RNA Extraction and Long Noncoding RNA Quantification

#### 5.4.1. RNA Extraction

Total RNA, including long noncoding RNA, was extracted from 200 μL of stored plasma using the miRNeasy Mini Kit (QIAGEN, GmbH, Hilden, Germany), strictly following the manufacturer’s instructions. The concentration and purity (A260/A280 ratio) of the extracted RNA were assessed using a NanoDrop spectrophotometer (Thermo Scientific, Waltham, MA, USA). RNA samples with an A260/A280 ratio between 1.8 and 2.1 were deemed acceptable. The extracted RNA was immediately stored at −80 °C until the reverse transcription step.

##### RNA Quality Metrics

**A.**  **RNA Concentration and Purity (NanoDrop):**

- A260/A280 ratio: Required range 1.8–2.1 (indicates protein contamination if <1.8)
- A260/A230 ratio: Required range 1.8–2.2 (indicates organic solvent contamination if <1.8)
- RNA concentration: Median 48.3 ng/µL (IQR: 35.7–62.8 ng/µL)
- Acceptance: 97.4% of samples met purity criteria on first extraction; 2.6% required re-extraction

**B.**  **RNA Integrity Assessment:**

RIN (RNA Integrity Number) values were NOT routinely measured for this study. We acknowledge this limitation and provide scientific justification:

Rationale for not requiring RIN:

- Serum/plasma RNA characteristics: Circulating cell-free RNA exists as short fragments (100–300 nucleotides) and exosome-protected molecules, NOT intact ribosomal RNA with 28S/18S peaks. RIN values designed for tissue RNA integrity (detecting 28S/18S ribosomal RNA degradation) do not apply to cell-free RNA in blood, which naturally lacks ribosomal RNA and appears “degraded” by RIN standards even when fully intact (RIN typically <3.0 for all serum samples regardless of quality).
- Alternative quality metrics used: A260/A280 purity ratios, successful RT efficiency (assessed by GAPDH Ct values), and RT-qPCR amplification curves demonstrating appropriate exponential amplification with single melt peaks (no primer dimers or non-specific products).
- Literature precedent: Published circulating lncRNA studies in HCC (Zheng et al., Lai et al., Sun et al.) [1,15,16] and other cancers uniformly report A260/A280 ratios without RIN values for serum/plasma samples, as RIN is inappropriate for cell-free RNA.

**C.**  **RNA Extraction Efficiency and RT Success:**

- GAPDH reference gene Ct values used as proxy for RNA quality and RT efficiency
- Acceptance criteria: GAPDH Ct 18–28 cycles (indicates adequate RNA input and successful reverse transcription)
- Observed GAPDH Ct: Median 22.4 (IQR: 20.8–24.3), all samples within acceptable range
- Samples with GAPDH Ct > 28 (indicating poor RNA quality/quantity or RT failure) were excluded and re-extracted (*n* = 4, 2.6%)

#### 5.4.2. Reverse Transcription (cDNA)

Complementary DNA (cDNA) was synthesized from the extracted RNA using the SensiFAST™ cDNA Synthesis Kit (Bioline, Brandenburg, Germany). The 20 μL reaction mixture consisted of 10 μL of RNA template, 4 μL of 5× TransAmp Buffer, 1 μL of Reverse Transcriptase enzyme, and 5 μL of Nuclease-free water. The reaction was carried out in an Applied Biosystems thermal cycler (Singapore) under the following conditions: 42 °C for 10 min (reverse transcription), followed by 95 °C for 5 min (enzyme inactivation), and a final hold at 4 °C. The synthesized cDNA was stored at −20 °C until quantitative PCR (qPCR) analysis.

#### 5.4.3. Quantitative Real-Time PCR (qPCR)

The expression levels of *MALAT1* and *UCA1* were quantified by real-time PCR using the SensiFAST™ SYBR No-ROX Kit (Bioline, Germany). The reaction was performed in a total volume of 20 μL, containing 10 μL of 2× SensiFAST SYBR mix, 0.8 μL of each forward and reverse primer (10 μM), 2 μL of cDNA template, and 6.4 μL of nuclease-free water. The primer sequences used were as follows:

- ***MALAT1***
  ○ Forward: 5′-CAG GCGTTGTGCGTAAGAGGA-3′
  ○ Reverse: 5′-TGCCGACCTCACGGATTTTT-3′
- ***UCA1***
  ○ Forward: 5′-CTCTCCATTGGGTTCACCATTC-3′
  ○ Reverse: 5′-GCGGGCAGGTTCTTAGAGGATGAG-3′
- ***GAPDH*** (Endogenous Control)
  ○ Forward: 5′-GTCAGCCGCATCTTCTTT-3′
  ○ Reverse: 5′-CGCCCATAGACCAAAT-3′

All RT-qPCR reactions were performed in duplicate (two technical replicates per sample). The mean Ct value of duplicates was used for quantification. Quality control criterion: Duplicate Ct values with a coefficient of variation (CV) >15% or Ct difference >0.5 cycles were excluded and repeated. Acceptance rate: 98.7% of samples passed on first run; 1.3% required repeat due to high inter-replicate variability.

The amplification was carried out on a Rotor-Gene Q real-time PCR cycler (QIAGEN, Germantown, MD, USA) with the following thermal profile: an initial denaturation at 95 °C for 2 min, followed by 40 cycles of denaturation at 95 °C for 5 s and annealing/extension at 60 °C for 30 s. A melt curve analysis was performed at the end of each run to confirm the specificity of the amplification products. All reactions were performed in duplicate. The relative expression levels of *MALAT1* and *UCA1* were calculated using the 2^−ΔΔCt^ method, with GAPDH as the endogenous reference gene.

The prognostic cut-off values (UCA1 >12.0 and MALAT1 >87.76, expressed as relative expression units calculated using the 2^−ΔΔCt^ method with GAPDH normalization) were derived from the external validation study by Abdelsattar et al. (2025) [14], which some of our research team co-authored. This study established these thresholds through rigorous receiver operating characteristic (ROC) curve analysis and optimized the Youden’s Index for predicting overall survival in a diagnostic cohort comprising HCC patients and chronic HCV controls (without HCC).

The established cut-offs also align with findings from independent validation studies:

- Zheng et al. (2017) [15] reported that serum UCA1 levels above their cohort-optimized threshold (approximately equivalent to our relative expression unit of 12.0) correlated with large tumor size, vascular invasion, advanced TNM stage, and served as an independent prognostic factor for poor overall survival (HR = 2.13, 95% CI: 1.45–3.12, *p* < 0.001).
- Lai et al. (2012) [16] demonstrated that tissue MALAT1 overexpression (using tissue-based cut-offs) predicted tumor recurrence after liver transplantation, establishing biological plausibility for circulating levels reflecting tumor aggressiveness.

### 5.5. Clinical Endpoint Definitions

#### 5.5.1. Treatment Response Assessment

Radiological response was evaluated using modified Response Evaluation Criteria in Solid Tumors (mRECIST) at Week 4 and Week 12. Response categories were defined as:

- **Good Response (GR)**: ≥30% decrease in the sum of viable (arterially enhancing) target lesion diameters
- **Partial Response (PR)**: ≥10% but <30% decrease
- **Stable Disease (SD)**: Neither sufficient shrinkage to qualify for PR nor sufficient increase to qualify for PD
- **Progressive Disease (PD)**: ≥20% increase in the sum of diameters, or appearance of new lesions

**Disease Control Rate (DCR)** was defined as the proportion of patients achieving GR, PR, or SD at first restaging (Week 4).

Time-to-Progression (TTP) and Overall Survival (OS).

- **Time-to-Progression (TTP)**: Interval from sorafenib initiation to radiological progression per mRECIST criteria. Patients who died without documented progression were censored at the last imaging assessment. TTP differs from Progression-Free Survival (PFS) in that death without progression is not counted as an event.
- **Overall Survival (OS)**: Interval from sorafenib initiation to death from any cause. Patients alive at study closure were censored at last follow-up.

#### 5.5.2. Resistance Pattern Classification

Resistance patterns based on temporal response dynamics:

**Primary (Innate) Resistance**: Progressive disease at first restaging (Week 4) with concurrent stable or rising biomarker levels from baseline. This phenotype reflects pre-existing biological resistance mechanisms. Primary (Innate) Resistance was defined as progressive disease (PD) at the first radiological restaging (Week 4) per mRECIST criteria.

Specific Criteria:

- **Timing:** First mRECIST assessment at Week 4 (4 weeks ± 3 days post-sorafenib initiation)
- **Radiological criterion:** ≥20% increase in sum of viable (arterially enhancing) target lesion diameters compared to baseline, OR appearance of new intrahepatic/extrahepatic lesions
- **Biomarker criterion:** UCA1 and/or MALAT1 levels at Week 4 remain stable (≤10% change) or increase (>10% elevation) compared to baseline, indicating absence of biomarker response paralleling radiological progression
- **Clinical interpretation:** Reflects pre-existing biological resistance mechanisms (intrinsic pathway alterations, constitutive drug efflux, baseline hypoxic tumor microenvironment) present before sorafenib exposure

**Acquired Resistance**: Initial disease control (GR/PR/SD at Week 4) followed by subsequent progression at Week 12 or later, accompanied by biomarker elevation (≥10% increase from nadir “lowest value achieved”). This pattern indicates the emergence of adaptive resistance during therapy.

Specific Criteria:

- **Week 4 response:** Good response (≥30% decrease in viable tumor), partial response (10–30% decrease), or stable disease (<10% change, no new lesions)
- Week 12 (or later) progression: ≥20% increase in viable tumor burden compared to Week 4 nadir, OR new lesions
- **Biomarker criterion:** UCA1 and/or MALAT1 levels at time of progression show ≥10% increase compared to nadir level (typically Week 4 or Week 8 for initial responders)
- **Clinical interpretation:** Adaptive resistance emergence during therapy (clonal selection, compensatory pathway activation, tumor microenvironment remodeling)

**Sustained Response**: Maintained disease control through Week 12 with stable or declining biomarker levels.

Specific Criteria:

- **Week 4 response:** Initial disease control (GR/PR/SD)
- **Week 12 status:** Continued disease control without progression
- **Biomarker criterion:** UCA1 and MALAT1 levels remain stable (≤10% fluctuation) or decline (>10% reduction) from baseline through Week 12
- Clinical interpretation: Effective sorafenib target inhibition, absence of intrinsic or adaptive resistance.

**Adverse Event Assessment and Dose Modifications.**

Adverse events (AEs) were graded according to Common Terminology Criteria for Adverse Events (CTCAE) version 5.0. Sorafenib dose reductions or temporary interruptions were permitted for Grade ≥3 toxicities:

**First occurrence**: Dose reduction to 400 mg once daily.

**Second occurrence**: Dose reduction to 400 mg every other day.

Persistent Grade 3 or any Grade 4 toxicity: Permanent discontinuation.

Patients who discontinued sorafenib due to toxicity before Week 4 were categorized as “discontinued due to toxicity” in the CONSORT diagram and excluded from analyses. The most common dose-limiting toxicities in this cohort were:

Hand-foot skin reaction (HFSR, Grade 3).

Diarrhea (Grade 3).

Hepatic decompensation (Grade 3–4).

Biomarker Elevation Lead Time.

The “biomarker-to-CT lead time” was calculated as the interval between the first documented biomarker elevation (≥10% increase from baseline or nadir) and subsequent radiological confirmation of progression by CT/MRI. This metric quantifies the early warning window that biomarker monitoring provides for clinical decision-making.

### 5.6. Statistical Analysis

Statistical analyses were performed using IBM SPSS Statistics (Version 26.0), MedCalc Statistical Software, and R software (Version 4.2.1). Categorical variables were presented as numbers (percentages) and compared using the Chi-square or Fisher’s exact test. Continuous variables were tested for normality using the Shapiro–Wilk test. Normally distributed data were expressed as mean ± standard deviation (SD) and compared using Student’s *t*-test or ANOVA. Non-normally distributed data were expressed as median (interquartile range) and compared using the Mann–Whitney U or Kruskal–Wallis test. Schoenfeld residual tests were computed using R software. LOWESS smoothing applied with span = 0.75. The proportional hazards assumption for the Cox models was verified using Schoenfeld residuals. Neither the global test nor any covariate-specific tests indicated significant violations (all *p*-values > 0.05; see Supplementary Table S7 and Figure 11).

Patients were stratified into ‘High’ and ‘Low’ expression groups using pre-validated prognostic cut-offs: UCA1 > 12.0 and MALAT1 > 87.76 (relative expression units, calculated using the 2^−ΔΔCt^ method with GAPDH normalization). These cut-off thresholds were derived from the external diagnostic validation study by Abdelsattar et al. (2025) [14], which established these values through receiver operating characteristic (ROC) curve analysis using Youden index optimization (J = sensitivity + specificity − 1) to maximize discriminative accuracy for distinguishing HCC from chronic HCV infection. In that validation cohort (N = 120: 60 HCC patients, 60 chronic HCV controls), MALAT1 >87.76 achieved an area under the curve (AUC) of 0.987 (95% CI: 0.971–0.998) with 91.7% sensitivity and 93.3% specificity, while UCA1 >12.0 achieved an AUC of 0.983 (95% CI: 0.965–0.996) with 88.3% sensitivity and 95.0% specificity. Critically, these thresholds also demonstrated prognostic validity in the sorafenib-treated subset of that cohort, where high expression correlated with significantly inferior survival outcomes (high UCA1: 50.0% vs. low UCA1: 78.3%, *p* = 0.018; high MALAT1: 47.6% vs. low MALAT1: 76.9%, *p* = 0.012).

Additional validation support derives from Zheng et al. (2017) [15], who identified similar UCA1 thresholds as independent prognostic factors for overall survival in HCC patients (HR = 2.13, 95% CI: 1.45–3.12, *p* < 0.001). We adopted these externally validated cut-offs as pre-specified stratification criteria (rather than optimizing thresholds within our dataset) to avoid optimization bias and overfitting, thereby ensuring that our prognostic estimates represent generalizable, unbiased effects rather than dataset-specific artifacts.

To validate these prespecified cut-offs in our therapeutic monitoring cohort, we performed an internal ROC analysis using 12-month mortality as the endpoint. The Youden-optimized cut-offs derived from our cohort were 11.8 for UCA1 (AUC = 0.648, 95% CI: 0.561–0.735) and 89.3 for MALAT1 (AUC = 0.672, 95% CI: 0.587–0.757), demonstrating remarkable concordance with the externally derived values—within 1.7% for UCA1 and 1.8% for MALAT1 (Supplementary Table S2). This cross-cohort concordance provides evidence that these thresholds can generalize across independent HCC populations and capture biologically meaningful risk stratification rather than cohort-specific artifacts.

The primary endpoints were Time-to-Progression (TTP) and Overall Survival (OS). The primary efficacy endpoints were Time-to-Progression (TTP) and Overall Survival (OS). TTP was defined as the time from the initiation of sorafenib treatment to radiological disease progression according to mRECIST criteria. For the TTP analysis, patients were not censored at the time of a dose reduction. This approach was taken to evaluate the proper time to loss of disease control for the intended therapeutic strategy, thereby separating the analysis of efficacy from that of tolerability. OS was calculated from treatment start until death from any cause. Survival curves were estimated using the Kaplan–Meier method and compared with the log-rank test. Univariable and multivariable Cox proportional hazards models were used to identify independent prognostic factors, with results reported as Hazard Ratios (HR) with 95% Confidence Intervals (CI). The predictive performance of biomarkers was assessed using time-dependent ROC analysis. A two-sided *p*-value < 0.05 was considered statistically significant for all analyses.

Patients were stratified independently for each biomarker. Consequently, the ‘Low’ and ‘High’ expression groups for UCA1 and MALAT1 are not mutually exclusive, allowing for the analysis of each lncRNA’s unique contribution.

## 6. Conclusions

This study provides evidence that UCA1 and MALAT1 are valuable “theragnostic biomarkers” for sorafenib-treated HCC. Baseline levels stratify patients by prognosis, while serial monitoring provides an early, non-invasive signal of treatment response and resistance, with a clinically meaningful lead time over standard imaging. MALAT1 demonstrates superior performance to UCA1. Integrating these lncRNAs into clinical decision-making can enable a more personalized, dynamic treatment approach, minimizing ineffective therapy and optimizing outcomes for patients with advanced HCC.

## Figures and Tables

**Table 1:** Baseline Demographics and Clinical Characteristics of the 154-Patient Cohort.

Characteristic	Value (N = 154)
Age (years), Mean ± SD	58.6 ± 11.0
Gender, *n* (%)	
**Male**	121 (78.6%)
**Female**	33 (21.4%)
Child-Pugh Class, *n* (%)	
**A**	73 (47.4%)
**B**	57 (37.0%)
**C**	24 (15.6%)
BCLC Stage, *n* (%)	
**B**	48 (31.2%)
**C**	106 (68.8%)
ECOG Performance Status, *n* (%)	
**0**	43 (27.9%)
**1**	85 (55.2%)
**2**	26 (16.9%)
Etiology (HCV Positive), *n* (%)	112 (72.7%)
Key Laboratory Values, Median (IQR)	
**AFP (ng/mL)**	54.3 (16.7–256.3)
**ALT (IU/L)**	39.0 (15.0–156.0)
**Albumin (g/dL)**	3.2 (1.8–4.5)

**Table 2:** Cross-Classification Matrix.

Characteristic Expression	MALAT1 Low	MALAT1 High	Total
**UCA1 Low**	13 (8.4%)	25 (16.2%)	34 (22.1%)
**UCA1 High**	28 (18.2%)	88 (57.1%)	120 (77.9%)
**Total**	41 (26.6%)	113 (73.4%)	154 (100%)

This table mathematically accounts for all 154 patients and demonstrates several key findings: Concordance Analysis: 65.6% of patients (101/154) exhibit concordant expression of both biomarkers, either both biomarkers low (*n* = 13) or both high (*n* = 88). This moderate-to-strong concordance indicates a positive correlation between UCA1 and MALAT1, which is biologically plausible given that both lncRNAs are upregulated in aggressive HCC and may share common transcriptional regulators, such as HIF-1α (hypoxia-inducible factor) and NF-κB (inflammatory signaling), as well as epigenetic modifiers responding to the tumor microenvironment.

**Table 3:** (**A**). Baseline Characteristics Stratified by UCA1 Expression. (**B**). Baseline Characteristics Stratified by MALAT1 Expression.

(A)			
Characteristic	UCA1 Low (≤12.0) (N = 34)	UCA1 High (>12.0) (N = 120)	*p*-Value
**UCA1 Expression, Median (IQR)**	8.1 (6.5–10.9)	27.8 (19.4–36.2)	<0.001
**Demographics**			
**Age (years), Median (IQR)**	58 (49–67)	59 (50–68)	0.841 *
**Male gender, *n* (%)**	30 (78.9)	91 (78.4)	0.947 **
**Clinical Stage and Liver Function**			
**Child–Pugh class, *n* (%)**			0.612 **
**A**	19 (50.0)	54 (46.6)	
**B**	14 (36.8)	43 (37.1)	
**C**	5 (13.2)	19 (16.3)	
**BCLC Stage C, *n* (%)**	25 (65.8)	81 (69.8)	0.632 **
**Etiology and Laboratory Values**			
**HCV Positive, *n* (%)**	29 (76.3)	83 (71.6)	0.561 **
**ALT (IU/L), Median (IQR)**	42 (25–70)	44 (26–73)	0.671 *
**AST (IU/L), Median (IQR)**	50 (32–78)	54 (34–82)	0.584 *
**Albumin (g/dL), Median (IQR)**	3.3 (3.0–3.7)	3.2 (2.9–3.6)	0.409 *
**Total Bilirubin (mg/dL), Median (IQR)**	2.8 (1.5–4.0)	3.1 (1.8–4.5)	0.287 *
**AFP (ng/mL), Median (IQR)**	68.5 (30.1–450.2)	49.8 (15.2–210.4)	0.483 *
**(B)**			
**Characteristic**	**MALAT1 Low (≤87.76) (N = 41)**	**MALAT1 High (>87.76) (N = 113)**	***p*** **-Value**
**MALAT1 Expression, Median (IQR)**	46.3 (31.4–64.7)	376.2 (252.8–518.5)	<0.001
**Demographics**			
**Age (years), Median (IQR)**	57 (47–67)	59 (50–69)	0.317 *
**Male gender, *n* (%)**	31 (75.6)	90 (79.6)	0.586 **
**Clinical Stage and Liver Function**			
**Child-Pugh class, *n* (%)**			**0.038** **
**A**	26 (63.4)	47 (41.6)	
**B**	13 (31.7)	44 (38.9)	
**C**	2 (4.9)	22 (19.5)	
**BCLC Stage C, *n* (%)**	28 (68.3)	78 (69.0)	0.930 **
**Etiology and Laboratory Values**			
**HCV Positive, *n* (%)**	28 (68.3)	84 (74.3)	0.452 **
**ALT (IU/L), Median (IQR)**	43 (25–72)	45 (26–75)	0.712 *
**AST (IU/L), Median (IQR)**	51 (30–78)	55 (33–83)	0.392 *
**Albumin (g/dL), Median (IQR)**	3.4 (3.1–3.8)	3.1 (2.8–3.5)	**0.017** *
**Total Bilirubin (mg/dL), Median (IQR)**	2.4 (1.3–3.6)	3.3 (1.9–4.7)	**0.009** *
**AFP (ng/mL), Median (IQR)**	45.3 (18.9–155.1)	62.5 (16.1–298.3)	0.324 *

Abbreviations: IQR, interquartile range; BCLC, Barcelona Clinic Liver Cancer; HCV, Hepatitis C Virus; ALT, alanine aminotransferase; AST, aspartate aminotransferase; AFP, alpha-fetoprotein. * *p*-value from Mann–Whitney U test. ** *p*-value from Chi-square test. Statistical significance set at *p* < 0.05.

**Table 4:** Disease Control Rate (DCR) at Week 4 by Biomarker Status.

Group	DC Events/Total N	DCR (%)	95% CI	*p*-Value
Overall Cohort	35/154	22.7%	(16.8–30.0%)	-
UCA1 High	21/116	17.5%	(11.7–25.3%)	0.007
UCA1 Low	14/38	36.8%	(22.6–53.4%)	
MALAT1 High	23/120	19.2%	(13.1–27.1%)	0.080 “borderline”
MALAT1 Low	12/34	35.3%	(21.5–52.1%)	

**Table 5:** Time-to-Progression (TTP) Stratified by Biomarker Status.

Group	N	Median TTP (Weeks)	95% CI	Log-Rank *p*-Value	Hazard Ratio (95% CI)
**Overall Cohort**	154	19.6	(19.3–22.7)	-	-
**UCA1 High**	120	18.0	(15.8–20.2)	0.002	1.67 (1.21–2.31)
**UCA1 Low**	34	21.9	(16.2–27.6)		Reference (HR = 1.00)
**MALAT1 High**	113	18.0	(15.9–20.1)	0.003	1.72 (1.24–2.38)
**MALAT1 Low**	41	25.2	(19.1–31.3)		Reference (HR = 1.00)

**Table 6:** Predictive Performance of Rising Biomarkers for Acquired Resistance.

Biomarker Pattern	Sensitivity	Specificity	PPV	NPV	Statistical Test (*p*-Value)
**UCA1 Rising (≥10%)**	97.2%	23.9%	75.5%	78.6%	*p* < 0.001
**MALAT1 Rising (≥10%)**	98.1%	21.7%	75.2%	83.3%	*p* < 0.001
**Either Biomarker Rising (≥10%)**	**99.1%**	**21.7%**	**74.8%**	**90.9%**	*p* < 0.001

A ≥10% increase in either UCA1 or MALAT1 from baseline to Week 12 was a highly sensitive predictor of acquired resistance. The combined rule (a rise in either) achieved the highest sensitivity (99.1%) for identifying patients who would progress, providing a substantial median lead time of 7.0 weeks before radiological confirmation. While specificity was modest, the high Negative Predictive Value (NPV) is clinically valuable, as the absence of a biomarker rise reliably identifies patients who will not develop resistance. Between-group comparisons for dose modifications were performed using Chi-square tests.

**Table 7:** Biomarker-to-CT Lead Time Analysis.

Lead-Time Parameter	Value
**Overall Cohort (N = 154)**	
**Mean ± SD (weeks)**	6.5 ± 3.0
**Median [IQR] (weeks)**	7.0 [5.0–8.8]
**Stratified by Week 12 Biomarker Status**	
**Either UCA1 or MALAT1 Rising (≥10%) (*n* = 143)**	7.0 [5.0–8.8]
**No Significant Biomarker Rise (*n* = 11)**	0.0 [0.0–1.2]
**Clinical Utility**	
**Patients with Actionable Lead-time (≥4 weeks)**	135 (87.7%)

Lead time is defined as the interval between a ≥10% biomarker elevation (at Week 12) and subsequent radiological confirmation of progression. The data demonstrate that rising biomarker levels provide a substantial, clinically actionable median lead time of 7 weeks, enabling a critical window for preemptive treatment modification in the majority of patients.

**Table 8:** Cox Proportional Hazards Model—Univariable and Multivariable Cox Regression Analysis for Time-to-Progression (TTP).

Variable	Category	Univariable Analysis	Multivariable Analysis		
		HR (95% CI)	*p*-Value	HR (95% CI)	*p*-Value
**UCA1**	High vs. Low	1.67 (1.21–2.31)	0.002	1.52 (1.09–2.12)	0.014
**MALAT1**	High vs. Low	1.72 (1.24–2.38)	0.003	1.61 (1.15–2.25)	**0.006**
**ECOG PS**	≥2 vs. 0–1	1.89 (1.31–2.72)	0.001	1.71 (1.18–2.48)	**0.005**
**Child-Pugh**	B vs. A	1.42 (1.01–1.99)	0.043	1.35 (0.96–1.90)	0.086
	C vs. A	2.31 (1.67–3.19)	<0.001	2.08 (1.49–2.91)	**<0.001**
**BCLC Stage**	C vs. B	1.56 (1.15–2.12)	0.004	1.43 (1.05–1.95)	0.024

HR: Hazard Ratio; CI: Confidence Interval; ECOG PS: Eastern Cooperative Oncology Group Performance Status. The multivariable model was adjusted for all variables listed in the table. Both biomarkers retain independent prognostic significance. Child-Pugh C and ECOG ≥ 2 show the strongest prognostic impact.

**Table 9:** Comparison of Prognostic Models.

Prognostic Model	Components	C-Index (95% CI)	AIC	Improvement over Base Model
**Base Clinical Model**	Child-Pugh + BCLC + ECOG	0.634 (0.581–0.687)	892	Reference
**Base + AFP**	Base + AFP ≥ 400 ng/mL	0.658 (0.606–0.710)	887	+0.024 (*p* = 0.18)
**Base + UCA1**	Base + UCA1 > 12.0	0.679 (0.628–0.730)	878	+0.045 (*p* = 0.04) *
**Base + MALAT1**	Base + MALAT1 > 87.76	0.694 (0.644–0.744)	871	+0.060 (*p* = 0.01) *
**Base + Both lncRNAs**	Base + UCA1 + MALAT1	0.701 (0.651–0.751)	868	+0.067 (*p* = 0.008) *
**Full Model**	All of the above	0.706 (0.657–0.755)	866	+0.072 (*p* = 0.005) *

C-index (Harrell’s concordance index) measures discriminative ability; values > 0.7 indicate good performance. AIC (Akaike Information Criterion): lower values indicate better model fit. * *p* < 0.05 indicates a significant improvement in discrimination. Clinical Interpretation: Adding MALAT1 to the base clinical model provides the greatest single-biomarker improvement in prognostic accuracy.

**Table 10:** Safety Profile and Correlation with Biomarker Status.

Parameter	Overall Cohort (N = 154)	High UCA1/MALAT1 (*n* = 120)	Low UCA1/MALAT1 (*n* = 34)	*p*-Value (High vs. Low)
**Adverse Events (Any Grade)**				
**Hand-Foot Skin Reaction (HFSR)**	87 (56.5%)	68 (56.7%)	19 (55.9%)	0.93
**Diarrhea**	73 (47.4%)	58 (48.3%)	15 (44.1%)	0.65
**Fatigue**	64 (41.6%)	51 (42.5%)	13 (38.2%)	0.64
**Hypertension**	41 (26.6%)	33 (27.5%)	8 (23.5%)	0.63
**Hepatic Decompensation**	9 (5.8%)	7 (5.8%)	2 (5.9%)	0.99
**Bleeding Events**	7 (4.5%)	6 (5.0%)	1 (2.9%)	0.60
**Grade 3–4 Adverse Events**				
**Any Grade ≥ 3 AE**	23 (14.9%)	19 (15.8%)	4 (11.8%)	0.52
**Hand-Foot Skin Reaction**	8 (5.2%)	7 (5.8%)	1 (2.9%)	0.48
**Diarrhea**	4 (2.6%)	3 (2.5%)	1 (2.9%)	0.89
**Hepatic Decompensation**	6 (3.9%)	5 (4.2%)	1 (2.9%)	0.73
**Bleeding Events**	2 (1.3%)	2 (1.7%)	0 (0.0%)	0.43
**Dose Modifications**				
**Patients Maintaining Full Dose**	112 (72.7%)	86 (71.7%)	26 (76.5%)	0.58
**Patients with ≥1 Dose Reduction**	30 (19.5%)	24 (20.0%)	6 (17.6%)	0.77
**Patients Discontinued due to Toxicity**	12 (7.8%)	10 (8.3%)	2 (5.9%)	0.65

Table 10 presents the safety analysis of the 154-patient cohort treated with sorafenib. Statistical comparisons using Chi-square tests between high- and low-UCA1/MALAT1 expression groups revealed no significant differences in dose-reduction rates (20.0% vs. 17.6%), (χ^2^ = 0.085, *p* = 0.77) or treatment discontinuation due to toxicity (8.3% vs. 5.9%), (χ^2^ = 0.206, *p* = 0.65), confirming that these biomarkers predict resistance rather than influence drug tolerability.

## Data Availability

All data generated or analyzed during this study are included in this published article.

## Supplementary Materials

The following supporting information can be downloaded at: https://www.mdpi.com/article/10.3390/ph19010070/s1, Table S1: Clinical and Statistical Terms; Table S2. Cut-Off Threshold Validation and Performance Characteristics for UCA1 and MALAT1 Biomarkers; Table S3: Distribution of Sorafenib Resistance Patterns in the Study Cohort; Figure S1: Kaplan–Meier Curves of Overall Survival Stratified by Sorafenib Resistance Pattern; Table S4: Primer Sequences and qPCR Reaction Protocol; Table S5: Complete Cox Proportional Hazards Models for Time-to-Progression; Table S6: Multiple Comparison Corrections for Primary and Secondary Endpoints; Table S7: Proportional Hazards Assumption Testing Results; Table S8: Sensitivity Analysis of Alternative Cut-off Strategies; Figure S2. Receiver operating characteristic (ROC) curves for lncRNA biomarker cut-off derivation and validation; Table S9: RT-qPCR Primer Sequences and Validation Metrics; Table S10: Dose Modification Analysis and Biomarker Independence; Table S11: Evidence-Based Actionable Interventions by Biomarker Status; Table S12: Baseline Characteristics by UCA1 and MALAT1 Expression Status; Table S13: Biomarker Correlation and Independence Analysis; Table S14: Comparative Performance of HCC Biomarkers; Table S15: Safety Profile and Adverse Event Analysis in Sorafenib-Treated HCC Cohort (N = 154).

## Abbreviations

AASLD	American Association for the Study of Liver Diseases
AFP	Alpha-fetoprotein
ALP	Alkaline Phosphatase
ALT	Alanine Aminotransferase
AST	Aspartate Aminotransferase
AUC	Area Under the Curve
BCLC	Barcelona Clinic Liver Cancer
cDNA	Complementary DNA
CI	Confidence Interval
CRP	C-reactive Protein
Ct	Cycle Threshold
DCR	Disease Control Rate
ECOG	Eastern Cooperative Oncology Group
ECL	Electrochemiluminescence
GGT	Gamma-Glutamyl Transferase
HCC	Hepatocellular Carcinoma
HCV	Hepatitis C Virus
HR	Hazard Ratio
INR	International Normalized Ratio
IQR	Interquartile Range
LnRNA = lncRNA	Long Noncoding RNA
MALAT1	Metastasis-Associated Lung Adenocarcinoma Transcript 1
OS	Overall Survival
qPCR	Quantitative Polymerase Chain Reaction
RECIST	Response Evaluation Criteria in Solid Tumors
ROC	Receiver Operating Characteristic
RT	Reverse Transcription
SD	Standard Deviation
TTP	Time-To-Progression
TLR4/NF-κB	Toll-like Receptor 4/Nuclear Factor Kappa B
UCA1	Urothelial Carcinoma-Associated 1

## References

[1] Sung H., Ferlay J., Siegel R.L., Laversanne M., Soerjomataram I., Jemal A., Bray F. (2021). Global cancer statistics 2020: GLOBOCAN estimates of incidence and mortality worldwide for 36 cancers in 185 countries. CA Cancer J. Clin..

[2] Llovet J.M., Kelley R.K., Villanueva A., Singal A.G., Pikarsky E., Roayaie S., Lencioni R., Koike K., Zucman-Rossi J., Finn R.S. (2021). Hepatocellular carcinoma. Nat. Rev. Dis. Primers.

[3] Llovet J.M., Ricci S., Mazzaferro V., Hilgard P., Gane E., Blanc J.F., de Oliveira A.C., Santoro A., Raoul J.L., Forner A. (2008). Sorafenib in advanced hepatocellular carcinoma. N. Engl. J. Med..

[4] Wilhelm S.M., Adnane L., Newell P., Villanueva A., Llovet J.M., Lynch M. (2008). Preclinical overview of sorafenib, a multikinase inhibitor that targets both Raf and VEGF and PDGF receptor tyrosine kinase signaling. Mol. Cancer Ther..

[5] Galle P.R., Forner A., Llovet J.M., Mazzaferro V., Piscaglia F., Raoul J.L., Schirmacher P., Vilgrain V. (2018). EASL Clinical Practice Guidelines: Management of hepatocellular carcinoma. J. Hepatol..

[6] Reig M., Forner A., Rimola J., Ferrer-Fàbrega J., Burrel M., Garcia-Criado Á., Kelley R.K., Galle P.R., Mazzaferro V., Salem R. (2022). BCLC strategy for prognosis prediction and treatment recommendation: The 2022 update. J. Hepatol..

[7] Cabral L.K.D., Tiribelli C., Sukowati C.H.C. (2020). Sorafenib resistance in hepatocellular carcinoma: The relevance of genetic heterogeneity. Cancers.

[8] Bartonicek N., Maag J.L.V., Dinger M.E. (2016). Long noncoding RNAs in cancer: Mechanisms of action and technological advancements. Mol. Cancer.

[9] Verma S., Sahu B.D., Mugale M.N. (2023). Role of LncRNAs in hepatocellular carcinoma. Life Sci..

[10] Hou Z.H., Xu X.W., Fu X.Y., Zhou L.D., Liu S.P., Tan D.M. (2020). Long noncoding RNA MALAT1 promotes angiogenesis and immunosuppressive properties of HCC cells by sponging miR-140. Am. J. Physiol. Cell Physiol..

[11] Wang Y., Mou Q., Zhu Z., Zhao L., Zhu L. (2021). MALAT1 promotes liver fibrosis by sponging miR-181a and activating TLR4-NF-κB signaling. Int. J. Mol. Med..

[12] Wang F., Ying H., He B., Pan Y., Liu X., Wang S., Gao S. (2015). Upregulated lncRNA-UCA1 contributes to progression of hepatocellular carcinoma through inhibition of miR-216b and activation of FGFR1/ERK signaling pathway. Oncotarget.

[13] Huang G., Li L., Liang C., Yu Y., He Y., Xu L., Liu Q. (2021). Upregulated UCA1 contributes to oxaliplatin resistance of hepatocellular carcinoma through inhibition of miR-138-5p and activation of AKT/mTOR signaling pathway. Pharmacol. Res. Perspect..

[14] Abdelsattar S., Basuni A.A., El-Abd M.G., Maksoud E.E.A., Sabry A., Mosbeh A., El-Hefnawy S.M., Darwish E., Aldesoky A.I., Elhanafy A.M. (2025). Diagnostic, prognostic, and therapeutic significance of long noncoding RNAs MALAT1 and UCA1 in HCV-complicated hepatocellular carcinoma. Egypt. J. Chem..

[15] Zheng Z., Guo X., Li J., Wang Z., Yang A., Zhao L., Zhou B., Chen R., Huang S. (2017). Serum long noncoding RNA urothelial carcinoma-associated 1: A novel biomarker for diagnosis and prognosis of hepatocellular carcinoma. J. Int. Med. Res..

[16] Lai M., Yang Z., Zhou L., Zhu Q., Xie H., Zhang F., Wu L., Chen L., Zheng S. (2012). Long non-coding RNA MALAT-1 overexpression predicts tumor recurrence of hepatocellular carcinoma after liver transplantation. Med. Oncol..

[17] Fan L., Huang X., Chen J., Zhang K., Gu Y.H., Sun J., Cui S.Y., Zhou Y. (2020). Long noncoding RNA MALAT1 contributes to sorafenib resistance by targeting miR-140-5p/Aurora-A signaling in hepatocellular carcinoma. Mol. Cancer Ther..

[18] Li J., Cui Z., Li H., Lv X., Gao M., Yang Z. (2018). Clinicopathological and prognostic significance of long noncoding RNA MALAT1 in human cancers: A review and meta-analysis. Cancer Cell Int..

[19] Nazih M., Khoder A.I., Waked I., El Senbawy M.S., Abdelsattar S., Badr Hassan S., Abdel-Latif M.M. (2025). Real-world outcomes and predictive factors in hepatocellular carcinoma patients treated with sorafenib: An 18-month ambispective cohort analysis. Future J. Pharm. Sci..

[20] Faul F., Erdfelder E., Lang A.G., Buchner A. (2007). G*Power 3: A flexible statistical power analysis program for the social, behavioral, and biomedical sciences. Behav. Res. Methods.

