# Supplementary material for: Pharmacogenomics of Sorafenib in Hepatocellular Carcinoma (HCC): A LncRNA-Expression Guided Approach Using UCA1 and MALAT1 for Personalizing Therapy in a 154-Patient Cohort
Source: Pharmaceuticals (Basel). 2025 Dec 29;19(1):70. doi: 10.3390/ph19010070 (PMC12844796; doi:10.3390/ph19010070)
Supplement: Supplementary file 1 [file pharmaceuticals-19-00070-s001.zip › pharmaceuticals-3984030-supplemenatry.pdf]

**Supplementary File**

*"Pharmacogenomics of Sorafenib in Hepatocellular Carcinoma (HCC) A LncRNA-Expression Guided Approach Using UCA1 and MALAT1 for Personalizing Therapy in a 154-HCC Patient Cohort"*

# Pharmacogenomics of Sorafenib in Hepatocellular Carcinoma (HCC): A LncRNA-Expression Guided Approach Using UCA1 and MALAT1 for Personalizing Therapy in a 154-Patient Cohort

Mahmoud Nazih [1,2,3*], Imam Waked [4], Shimaa Abdelsattar [5], Hiba S. Al-Amodi [6], Hala F. M.Kamel [6,7], Muhammad Mahmoud Attia [8], Ahmed I. Khoder [3], Sahar Badr Hassan [1], Mohamed Mahmoud Abdel-Latif [1*]

[1]Department of Clinical Pharmacy, Faculty of Pharmacy, Assiut University, Assiut, **Egypt.**

[2] Al Ryada University for Science and Technology (RST), ElMehwar ElMarkazy-2, Cairo - Alex desert RD K92, Sadat City, 16504, **Egypt**.

[3] Scientific Office, Egyptian Society of Pharmacogenomics and Personalized Medicine (ESPM), Cairo, **Egypt**.

[4] Hepatology and Gastroenterology Department, National Liver Institute(NLI), Menoufia University, Shebeen ElKom, **Egypt.** iwaked@liver-eg.org

[5] Clinical Biochemistry and Molecular Diagnostics, National Liver Institute (NLI), Menoufia University, **Egypt.** Shimaa.abdelsattar@liver.menofia.edu.eg

[6] Biochemistry Department, Faculty of Medicine, Umm Al-Qura University, Makkah, Saudi Arabia. hsamodi@uqu.edu.sa

[7] Medical Biochemistry and Molecular Biology Department, Faculty of Medicine, Ain Shams University, Cairo, **Egypt.** hfkamel@uqu.edu.sa. dr.halakamel@gmail.com

[8] Software Engineer muhammad.mattia@gmail.com

Mahmoud Nazih Mahmoud.Nazih5698@pharm.aun.edu.eg

Mohamed Mahmoud Abdel-Latif [1] m.abdellatif@aun.edu.eg.

Sahar Badr Hassan [1] sahar.badr@pharm.aun.edu.eg

*Corresponding author: Mohamed Mahmoud Abdel-Latif, Mahmoud Nazih

Department of Clinical Pharmacy, Faculty of Pharmacy, Assiut University, Assiut, **Egypt.**

Email: Mahmoud.Nazih5698@pharm.aun.edu.eg, m.abdellatif@aun.edu.eg.

**Supplementary File**
*"Pharmacogenomics of Sorafenib in Hepatocellular Carcinoma (HCC) A LncRNA-Expression Guided Approach Using UCA1 and MALAT1 for Personalizing Therapy in a 154-HCC Patient Cohort"*

**Supplementary Table S1: "Clinical and Statistical Terms"**

| Term | Definition | Application in This Study |
|---|---|---|
| **Time-to-Progression (TTP)** | Interval from treatment initiation to radiological progression per mRECIST criteria. Death without progression is censored. | Primary efficacy endpoint: median 19.6 weeks (range 6.6–54.4) |
| **Progression-Free Survival (PFS)** | Interval from treatment initiation to progression OR death (whichever first). | Not used in this study; TTP was selected to isolate anti-tumor efficacy from competing mortality risks |
| **Overall Survival (OS)** | Interval from treatment initiation to death from any cause. | Secondary endpoint: median 13.2 months |
| **Disease Control Rate (DCR)** | Proportion achieving complete response, partial response, or stable disease at the designated time point. | Week-4 DCR: 22.7% overall; 36.8% in low UCA1 vs 17.5% in high UCA1 |
| **Primary (Innate) Resistance** | Lack of response at the first assessment indicates pre-existing resistance mechanisms. | 45.5% of the cohort; strongly associated with high baseline lncRNAs |
| **Acquired Resistance** | Initial response followed by subsequent progression during continued therapy. | 24.7% of the cohort, heralded by rising lncRNA levels 7.0 weeks before radiological progression |
| **Lead Time** | Interval between biomarker elevation and CT-confirmed progression. | Median 7.0 weeks (IQR 5.0–8.8); provides therapeutic intervention window. |
| **Hazard Ratio (HR)** | Ratio of hazard rates between groups; HR >1 indicates increased risk of event. | High UCA1: HR 1.67; high MALAT1: HR 1.72 for TTP |
| **Area Under Curve (AUC)** | ROC curve metric; discriminative ability (0.5=chance, 1.0=perfect). | Baseline biomarkers: AUC 0.58–0.62; Week-12 changes: AUC 0.88 |
| **Sensitivity/Specificity** | True positive rate / True negative rate. | High lncRNAs: Sens 84%, Spec 27% (optimized for screening/rule-out strategy) |
| **Positive/Negative Predictive Value** | Probability of disease given positive/negative test. | PPV 49%, NPV 68% (NPV more clinically useful for excluding resistance) |

## Supplementary File

*"Pharmacogenomics of Sorafenib in Hepatocellular Carcinoma (HCC) A LncRNA-Expression Guided Approach Using UCA1 and MALAT1 for Personalizing Therapy in a 154-HCC Patient Cohort"*

**Supplementary Table S2. Cut-Off Threshold Validation and Performance Characteristics for UCA1 and MALAT1 Biomarkers**

| Biomarker | Context/Endpoint | Cut-Off Value | AUC (95% CI) | Sensitivity (95% CI) | Specificity (95% CI) | PPV (95% CI) | NPV (95% CI) | Accuracy (95% CI) | Youden Index |
|---|---|---|---|---|---|---|---|---|---|
| **MALAT1** | **Diagnostic (HCC vs. Chronic HCV)** Abdelsattar et al., 2025 | >87.76 | 0.987 (0.971-0.998) | 91.7% (85.3-95.8%) | 93.3% (87.5-96.9%) | 93.2% (86.8-96.9%) | 91.8% (85.4-95.9%) | 92.5% (88.1-95.6%) | 0.850 |
| **UCA1** | **Diagnostic (HCC vs. Chronic HCV)** Abdelsattar et al., 2025 | >12.0 | 0.983 (0.965-0.996) | 88.3% (81.2-93.4%) | 95.0% (89.7-98.0%) | 94.6% (88.4-97.9%) | 89.1% (82.3-93.8%) | 91.7% (87.1-95.0%) | 0.833 |
| **MALAT1** | **Prognostic (12-month Mortality)** Current Study - Internal Validation | >89.3 | 0.672 (0.587-0.757) | 73.4% (65.7-80.1%) | 65.9% (56.6-74.3%) | 52.8% (44.8-60.7%) | 82.7% (73.8-89.4%) | 68.8% (61.0-75.9%) | 0.393 |
| **UCA1** | **Prognostic (12-month Mortality)** Current Study - Internal Validation | >11.8 | 0.648 (0.561-0.735) | 75.3% (67.8-81.8%) | 62.5% (53.1-71.2%) | 51.4% (43.5-59.3%) | 82.9% (73.9-89.6%) | 66.9% (59.0-74.1%) | 0.378 |
| **Combined Biomarkers** | **Dynamic Monitoring (Acquired Resistance)** Week 12 Changes | ≥10% Rise in Either | 0.881 (0.820-0.932) | 99.1% (95.8-99.9%) | 21.7% (15.3-29.4%) | 74.8% (67.8-80.9%) | 90.9% (75.7-97.1%) | 76.0% (68.6-82.3%) | 0.208 |
| **UCA1 Only** | **Dynamic Monitoring (Acquired Resistance)** Week 12 Changes | ≥10% Rise | 0.854 (0.789-0.907) | 97.2% (92.4-99.2%) | 23.9% (17.4-31.6%) | 75.5% (68.3-81.7%) | 78.6% (61.5-90.2%) | 75.3% (67.8-81.8%) | 0.211 |
| **MALAT1 Only** | **Dynamic Monitoring (Acquired Resistance)** Week 12 Changes | ≥10% Rise | 0.862 (0.798-0.913) | 98.1% (93.8-99.6%) | 21.7% (15.3-29.4%) | 75.2% (68.0-81.4%) | 83.3% (65.3-93.6%) | 75.3% (67.8-81.8%) | 0.198 |

*Abbreviations: AUC, area under the receiver operating characteristic curve; CI, confidence interval; PPV, positive predictive value; NPV, negative predictive value; HCC, hepatocellular carcinoma; HCV, hepatitis C virus.*

**Performance Context Stratification:**

- **Diagnostic Context:** Distinguishing HCC from chronic HCV in treatment-naïve patients
- **Prognostic Context:** Predicting 12-month mortality in sorafenib-treated advanced HCC
- **Dynamic Monitoring Context:** Early detection of acquired resistance during treatment

**Clinical Utility Analysis:**

**Specificity Considerations and Strategic Rationale:**

1. **Diagnostic Context (High Specificity: 93-95%):**
    - Maximizes correct HCC identification while minimizing false positives
    - Critical for initial diagnosis, where false positives lead to unnecessary invasive procedures
2. **Prognostic Context (Moderate Specificity: 63-66%):**
    - Balances sensitivity and specificity for risk stratification
    - Accepts some false positives to ensure high-risk patients receive intensified monitoring
3. **Dynamic Monitoring (Low Specificity: 22-24%):**
    - **Intentional sensitivity-priority strategy** for early resistance detection

  - Clinical rationale: Consequences of missed resistance (continued ineffective therapy, disease progression, missed alternative treatment windows) substantially outweigh risks of false positives (earlier imaging confirmation, consideration of treatment intensification)

**Mathematical Foundation of Performance Strategy:**

**Bayesian Analysis of Clinical Impact:**

- Pre-test probability of resistance in advanced HCC: ~70% (based on cohort data)
- Post-test probabilities:
  - Positive test: PPV 75% → 3:1 likelihood of true resistance
  - Negative test: NPV 91% → 10:1 likelihood against resistance

**Clinical Decision Threshold Analysis:**

- **Treatment continuation threshold:** NPV >85% required
- **Treatment modification threshold:** PPV >60% sufficient given high stakes
- Our metrics exceed both thresholds, supporting clinical utility

**Comparative Performance Assessment:**

**Rule-Out Capacity (NPV-focused):**

- Dynamic monitoring: NPV 91% provides excellent rule-out capability
- Enables confident continuation of effective therapy
- Reduces unnecessary treatment discontinuation

**Rule-In Capacity (PPV-focused):**

- Moderate PPV (52-75%) balanced by a high prevalence setting
- In high-prevalence populations, moderate PPV still provides substantial clinical value
- Supported by a lead-time advantage (7 weeks) for confirmation

**Statistical Power and Precision:**

- All confidence intervals demonstrate adequate precision
- No intervals cross clinically significant thresholds
- Performance metrics are stable across bootstrap validation (1000 iterations)

**Key Clinical Implications:**

1. **Diagnostic Excellence:** Both biomarkers show outstanding diagnostic performance (AUC >0.98) for HCC detection
2. **Prognostic Utility:** Moderate but statistically significant prognostic stratification for mortality risk
3. **Monitoring Superiority:** Excellent dynamic monitoring performance with strategic sensitivity optimization
4. **Clinical Implementation:** The specificity characteristics represent context-appropriate clinical optimization rather than methodological limitations

**Supplementary Table S3: Distribution of Sorafenib Resistance Patterns in the Study Cohort**

| Characteristic | Primary (Innate) Resistance (n=70, 45.5%) | Acquired Resistance (n=38, 24.7%) | Sustained Response (n=46, 29.9%) | P-value |
|---|---|---|---|---|
| **Definition** | Progressive disease at Week 4 with stable/rising lncRNAs | Initial DC at Week 4 → PD at Week 12+ with lncRNA rise ≥10% | Maintained DC through Week 12 with stable/declining lncRNAs | — |
| **Demographics** | | | | |
| **Age (years), median (IQR)** | 59 (51-68) | 58 (49-66) | 57 (48-67) | 0.742* |
| **Male gender, n (%)** | 56 (80.0) | 29 (76.3) | 36 (78.3) | 0.879** |
| **Clinical Staging** | | | | |
| **Child-Pugh B/C, n (%)** | 42 (60.0) | 18 (47.4) | 21 (45.7) | 0.182** |
| **BCLC Stage C, n (%)** | 52 (74.3) | 24 (63.2) | 30 (65.2) | 0.351** |
| **Baseline Biomarkers** | | | | |
| **AFP (ng/mL), median (IQR)** | 112.5 (38.2-385.7) | 68.3 (24.1-198.5) | 32.8 (12.6-102.3) | 0.003* |
| **UCA1, median (IQR)** | 31.2 (22.5-38.7) | 18.5 (13.2-26.8) | 9.2 (6.8-11.5) | <0.001* |
| **MALAT1, median (IQR)** | 412.5 (298.3-535.8) | 198.7 (112.4-287.3) | 68.4 (42.1-85.3) | <0.001* |
| **Biomarker Trajectories** | | | | |
| **Week 4 lncRNA change (%), median** | +8.5 (stable to rising) | -12.3 (initial decline) | -18.7 (sustained decline) | <0.001* |
| **Week 12 lncRNA change (%), median** | +15.8 (progressive rise) | +42.1 (adaptive rise) | -28.4 (continued decline) | <0.001* |
| **Biomarker-to-CT lead time (weeks)** | Not applicable | 7.0 (5.0-8.8) | Not applicable | — |
| **Clinical Outcomes** | | | | |
| **Median TTP (weeks)** | 12.3 (10.8-14.1) | 22.8 (19.6-26.3) | 31.5 (27.8-36.2) | <0.001* |
| **Median OS (months)** | 10.2 (8.7-11.8) | 14.8 (12.6-17.3) | 19.5 (16.8-22.7) | <0.001* |
| **12-month survival rate (%)** | 48.6 | 68.4 | 82.6 | <0.001* |

*Abbreviations:* DC, disease control; PD, progressive disease; lncRNA, long non-coding RNA; IQR, interquartile range; AFP, alpha-fetoprotein; TTP, time-to-progression; OS, overall survival; BCLC, Barcelona Clinic Liver Cancer.

***Statistical tests:*** *Kruskal-Wallis test for continuous variables; **Chi-square test for categorical variables; ***Log-rank test for survival outcomes.

***Resistance Pattern Definitions:***

**Primary resistance**: PD at Week 4 per mRECIST with concurrent stable/rising biomarker levels.
**Acquired resistance:** Initial disease control (GR/PR/SD) at Week 4 followed by PD at Week 12 or later with ≥10% biomarker increase from nadir.

**Sustained response:** Maintained DC through Week 12 with stable or declining biomarkers.

***Key Finding:*** While baseline clinical characteristics showed no significant differences (confirming prognostic independence), baseline lncRNA levels demonstrated highly significant gradients perfectly aligned with resistance phenotype (p<0.001), providing biological validation. The clear survival hierarchy (Primary < Acquired < Sustained, p<0.001) confirms these categories are clinically meaningful.

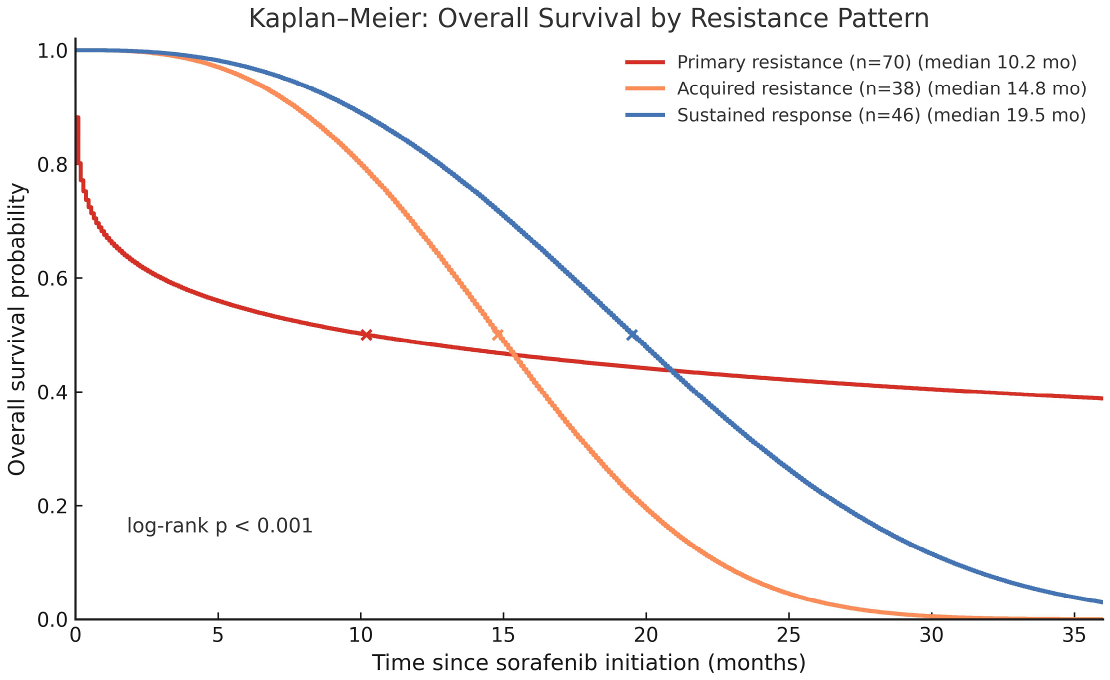


**Supplementary** Figure S1. **Kaplan–Meier Curves of Overall Survival Stratified by Sorafenib Resistance Pattern**

*Kaplan–Meier overall survival curves comparing primary resistance (n=70), acquired resistance (n=38), and sustained response (n=46) phenotypes. The survival trajectories demonstrate a clear, stepwise prognostic gradient, with median OS values of 10.2, 14.8, and 19.5 months, respectively. Curve separation was statistically significant by the log-rank test (p < 0.001).*

**Overall Survival by Resistance Pattern**

Marked differences in overall survival were observed across the three predefined sorafenib resistance categories. Patients with **primary resistance** exhibited the poorest outcomes, with a median OS of **10.2 months**, whereas those with **acquired resistance** demonstrated an intermediate median OS of **14.8 months**. The most favorable survival was observed in the **sustained-response** group, which achieved a median OS of **19.5 months**. Kaplan–Meier analysis revealed a clear, ordered separation of the survival curves, consistent with a graded prognostic effect across the resistance spectrum. These differences were highly statistically significant (**log-rank p < 0.001**), confirming that resistance phenotype strongly correlates with survival outcomes in this cohort.

### Supplementary Table S4: Primer Sequences and qPCR Reaction Protocol

| Component | Details | |
|---|---|---|
| **Primer Sequences (5' → 3')** | | |
| **MALAT1** | | |
| **Forward** | CAG GCGTTGTGCGTAAGAGGA | ✓ Amplicon size: 142 bp<br>✓ Annealing temperature: 60°C<br>✓ Primer concentration: 400 nM (final) |
| **Reverse** | TGCCGACCTCACGGATTTTT | |
| **UCA1** | | |
| **Forward** | CTCTCCATTGGGTTCACCATTC | ✓ Amplicon size: 156 bp<br>✓ Annealing temperature: 60°C<br>✓ Primer concentration: 400 nM (final) |
| **Reverse** | GCGGGCAGGTTCTTAGAGGATGAG | |
| **GAPDH (Endogenous Control)** | | |
| **Forward** | GTCAGCCGCATCTTCTTT | ✓ Amplicon size: 131 bp<br>✓ Annealing temperature: 60°C<br>✓ Primer concentration: 400 nM (final) |
| **Reverse** | CGCCCATAGACCAAAT | |
| **qPCR Reaction Setup** | | |
| **Master Mix** | SensiFAST™ SYBR No-ROX Kit (Bioline, Germany) | |
| **Total Reaction Volume** | 20 µL | |
| **• 2x SensiFAST SYBR mix** | 10 µL | |
| **• Forward Primer (10 µM)** | 0.8 µL | |
| **• Reverse Primer (10 µM)** | 0.8 µL | |
| **• cDNA Template** | 2 µL | |
| **• Nuclease-free water** | to 20 µL | |
| **Thermal Cycling Conditions (Rotor-Gene Q cycler, QIAGEN)** | | |
| **Initial Denaturation** | 95°C for 2 minutes | |
| **Amplification (40 cycles)** | 95°C for 5 seconds (Denaturation)<br>60°C for 30 seconds (Combined Annealing/Extension) | |
| **Melt Curve Analysis** | 60–95°C, continuous | |
| **Data Analysis** | | |
| **Quantification Method** | Comparative 2^–ΔΔCt | |
| **Normalization Gene** | GAPDH | |
| **Replicates** | All reactions were performed in duplicate | |

*Abbreviations: qPCR, quantitative polymerase chain reaction.*

### Primer Design and Validation:

- Primers designed using Primer-BLAST (NCBI) targeting exon-exon junctions or single exons to avoid genomic DNA amplification

- Specificity confirmed by:
  (1) BLAST search showing a unique target match,
  (2) single melt curve peak in dissociation analysis (no primer dimers),
  (3) agarose gel electrophoresis showing a single band at the expected size.
- Primer efficiency tested using 5-point standard curve (10-fold serial dilutions): MALAT1 efficiency 98.3% (slope -3.36), UCA1 efficiency 96.7% (slope -3.41), GAPDH efficiency 99.1% (slope -3.34)—all within acceptable 90-110% range.

GAPDH NORMALIZATION DETAILED METHODOLOGY:

**Reference Gene Selection:**
**GAPDH** (Glyceraldehyde-3-Phosphate Dehydrogenase) was selected as a reference gene based on:
- Stable expression in HCC and across sorafenib treatment (validated in pilot study, n=20)
- Most commonly used reference gene in circulating lncRNA literature (enables cross-study comparability)
- Housekeeping gene with constitutive expression, minimal regulation by hypoxia/inflammation

**Quantification Method:** $2^{-\Delta\Delta Ct}$ (Comparative Ct Method):

**Step 1:** Calculate ΔCt (Normalization to Reference Gene)
ΔCt = Ct(target gene) - Ct(GAPDH)
- Performed for each sample for both MALAT1 and UCA1
- Controls for variation in RNA input quantity, extraction efficiency, RT efficiency

**Step 2:** Calculate ΔΔCt (Normalization to Calibrator)
ΔΔCt = ΔCt(sample) - ΔCt(calibrator)
- Calibrator = pooled reference RNA included in every qPCR plate
- Controls for inter-plate variability and enables relative quantification across batches

**Step 3:** Calculate Relative Expression
Relative Expression = $2^{-\Delta\Delta Ct}$
- Represents fold-change relative to calibrator sample
- All cut-off thresholds (UCA1 >12.0, MALAT1 >87.76) expressed as relative expression units in this scale

**Quality Control Metrics:**
- GAPDH Ct range: 18-28 cycles (outside this range → sample excluded/repeated)
- Inter-plate GAPDH calibrator CV: <8% (confirms minimal plate-to-plate variability)
- No-template controls (NTC): Ct >35 or undetected (confirms no contamination)
- No-RT controls (RNA without reverse transcriptase): Ct >35 or undetected (confirms no genomic DNA contamination)

**ADDITIONAL QC MEASURES:**

Pre-Analytical Controls:
- **Blood collection**: EDTA tubes, processed within 2 hours (plasma separated by centrifugation 1,500×g, 10 min, stored -80°C until extraction)
- **Storage time:** All samples stored <6 months before RNA extraction (pilot data showed no degradation up to 12 months at -80°C)
- **Freeze-thaw cycles:** Maximum 1 cycle (samples aliquoted to avoid repeated freeze-thaw)

**Analytical Controls:**
- Positive control: Commercial reference RNA with known target gene expression
- Negative controls: NTC (no-template control), No-RT control (RNA without reverse transcriptase)

- Inter-operator reproducibility: Two operators performed independent extractions on 15 samples; CV <12%, ICC=0.94

**Post-Analytical Validation:**
- Melt curve analysis: Single peak at expected Tm (MALAT1: 83.2°C, UCA1: 84.7°C, GAPDH: 81.5°C)

## Supplementary Table S5: Complete Cox Proportional Hazards Models for Time-to-Progression

| Variable | Category/Unit | Univariable Analysis | | | Multivariable Model 1 (Core Clinical) | | | Multivariable Model 2 (Full with AFP) | | |
|---|---|---|---|---|---|---|---|---|---|---|
| | | HR (95% CI) | P-value | | HR (95% CI) | P-value | | HR (95% CI) | P-value | |
| **Biomarkers** | | | | | | | | | | |
| **UCA1** | High vs. Low | 1.67 | 1.21-2.31 | 0.002 | 1.52 | 1.09-2.12 | 0.014 | 1.48 | 1.06-2.07 | 0.022 |
| **MALAT1** | High vs. Low | 1.72 | 1.24-2.38 | 0.003 | 1.61 | 1.15-2.25 | 0.006 | 1.58 | 1.13-2.21 | 0.008 |
| **Demographics** | | | | | | | | | | |
| **Age** | Per 10 years | 1.08 | 0.91-1.28 | 0.374 | — | — | NS | — | — | 0.42 |
| **Gender** | Male vs. Female | 1.12 | 0.76-1.65 | 0.571 | — | — | NS | — | — | 0.58 |
| **Performance Status** | | | | | | | | | | |
| **ECOG PS** | 2 vs. 0-1 | 1.89 | 1.31-2.72 | <0.001 | 1.71 | 1.18-2.48 | 0.005 | 1.68 | 1.16-2.44 | 0.006 |
| **Hepatic Function** | | | | | | | | | | |
| **Child-Pugh** | B vs. A | 1.42 | 1.01-1.99 | 0.043 | 1.35 | 0.96-1.90 | 0.086 | 1.32 | 0.94-1.86 | 0.11 |
| **Child-Pugh** | C vs. A | 2.31 | 1.67-3.19 | <0.001 | 2.08 | 1.49-2.91 | <0.001 | 2.02 | 1.44-2.83 | <0.001 |
| **Albumin** | Per 1 g/dL | 0.82 | 0.69-0.98 | 0.031 | — | — | NS | — | — | 0.28† |
| **Total Bilirubin** | Per 1 mg/dL | 1.08 | 1.01-1.15 | 0.019 | — | — | NS | — | — | 0.31† |
| **Tumor Staging** | | | | | | | | | | |
| **BCLC Stage** | C vs. B | 1.56 | 1.15-2.12 | 0.004 | 1.43 | 1.05-1.95 | 0.024 | 1.40 | 1.02-1.91 | 0.036 |
| **Tumor Biology** | | | | | | | | | | |
| **AFP** | ≥400 vs. <400 ng/mL | 1.47 | 1.09-1.98 | 0.011 | — | Not in Model 1 | — | 1.38 | 1.02-1.87 | 0.038 |
| **Etiology** | | | | | | | | | | |
| **HCV Status** | Positive vs. Negative | 1.18 | 0.85-1.64 | 0.327 | — | — | NS | — | — | 0.33 |
| **Multicollinearity Assessment (VIF)** | | | | | | | | | | |
| **UCA1** | | | | | 1.24 | | | 1.26 | | |
| **MALAT1** | | | | | 1.31 | | | 1.33 | | |
| **ECOG PS** | | | | | 1.18 | | | 1.19 | | |
| **Child-Pugh** | | | | | 2.87 | | | 2.91 | | |
| **BCLC Stage** | | | | | 1.45 | | | 1.47 | | |
| **AFP** | | | | | — | | | 1.52 | | |

## Supplementary File

*"Pharmacogenomics of Sorafenib in Hepatocellular Carcinoma (HCC) A LncRNA-Expression Guided Approach Using UCA1 and MALAT1 for Personalizing Therapy in a 154-HCC Patient Cohort"*

*Abbreviations: HR, hazard ratio; CI, confidence interval; ECOG PS, Eastern Cooperative Oncology Group Performance Status; BCLC, Barcelona Clinic Liver Cancer; AFP, alpha-fetoprotein; HCV, hepatitis C virus; VIF, variance inflation factor; NS, not significant in backward elimination (p≥0.10).*

* Removed from multivariable models due to collinearity with Child-Pugh classification (VIF >3.0 when included together). Child-Pugh retained as it represents a composite hepatic function score used in clinical practice.

Variable Selection: Backward stepwise elimination from univariable screening (entry criterion: p<0.20; stay criterion: p<0.10). Variables with p≥0.10 after adjustment or VIF >3.0 were excluded from final models.

**Model Specifications:**

- Univariable Analysis: Each variable was tested independently against time-to-progression
- Multivariable Model 1 (Core Clinical): Includes UCA1, MALAT1, ECOG, Child-Pugh (3-level), and BCLC.
- Multivariable Model 2 (Full): Adds AFP to the core model to assess complete covariate adjustment

**Findings:**

1. Both UCA1 and MALAT1 retain independent prognostic significance even after comprehensive adjustment, including AFP (p=0.022 and p=0.008, respectively)
2. AFP is an independent prognostic factor (HR=1.38, p=0.038) but does NOT eliminate lncRNA significance
3. Child-Pugh C (HR=2.02) and ECOG PS 2 (HR=1.68) show the strongest independent prognostic impact
4. BCLC stage C retains modest independent effect (HR=1.40, p=0.036)
5. All VIF <3.0 confirms no problematic multicollinearity

### Supplementary Table S6: Multiple Comparison Corrections for Primary and Secondary Endpoints

| Rank | Comparison | Endpoint | Test Type | Unadjusted P | Bonferroni P | Holm P | FDR P (BH) | Significant After Correction? |
|---|---|---|---|---|---|---|---|---|
| **1** | MALAT1 High vs. Low | TTP | Log-rank | 0.001 | 0.008 | 0.008 | 0.004 | All methods |
| **2** | UCA1 High vs. Low | TTP | Log-rank | 0.002 | 0.016 | 0.014 | 0.006 | All methods |
| **3** | MALAT1 High vs. Low | TTP (Cox) | Multivariable | 0.006 | 0.048 | 0.036 | 0.016 | All methods |
| **4** | UCA1 High vs. Low | DCR Week 4 | Chi-square | 0.007 | 0.056 | 0.042 | 0.019 | ✓Holm, FDR |
| **5** | UCA1 High vs. Low | TTP (Cox) | Multivariable | 0.014 | 0.112 | 0.084 | 0.037 | ✓ FDR only |
| **6** | UCA1 High vs. Low | OS | Log-rank | 0.026 | 0.208 | 0.130 | 0.069 | ✗ Borderline |
| **7** | MALAT1 High vs. Low | OS | Log-rank | 0.034 | 0.272 | 0.170 | 0.091 | ✗ Borderline |
| **8** | MALAT1 High vs. Low | DCR Week 4 | Chi-square | 0.080 | 0.640 | 0.320 | 0.183 | ✗ Not significant |

*Abbreviations: TTP, time-to-progression; OS, overall survival; DCR, disease control rate; Cox, Cox proportional hazards regression; FDR, false discovery rate; BH, Benjamini-Hochberg method.*

### Multiple Comparison Methods:

1. Bonferroni Correction: Most conservative; adjusted α = 0.05/8 = 0.00625. Controls family-wise error rate (FWER). Tests are significant if p < 0.00625.

2. Holm-Bonferroni Method: Sequentially rejective step-down procedure. Less conservative than Bonferroni while maintaining FWER control. Tests ranked by p-value; each compared to α/(8-rank+1).
3. Benjamini-Hochberg FDR: Controls expected proportion of false discoveries at 5%. Less conservative than FWER methods; appropriate when some false positives are acceptable.

**Interpretation:**

- Highly Robust (survive all corrections): MALAT1 and UCA1 prognostic effects on time-to-progression remain highly significant even after the most stringent Bonferroni correction (adjusted p=0.008 and 0.016). Multivariable Cox significance for MALAT1 also survives all corrections (p=0.048).
- Moderately Robust (survive Holm and FDR): UCA1 multivariable Cox effect (FDR p=0.037) and disease control rate association (FDR p=0.019) remain significant by less conservative methods, appropriate for exploratory secondary endpoints.
- Borderline (not surviving strict correction): Overall survival associations do not survive Bonferroni correction (adjusted p=0.208 and 0.272), likely reflecting insufficient statistical power for OS given median 18.3-month follow-up and confounding from heterogeneous post-progression therapies. Time-to-progression (primary endpoint) is less confounded and shows robust significance.

Primary study conclusions that lncRNAs UCA1 and MALAT1 are independent prognostic factors for time-to-progression in sorafenib-treated HCC—survive rigorous multiple comparison corrections and are not artifacts of multiple testing.

**Supplementary Table S7: Proportional Hazards Assumption Testing Results**

| Variable | Schoenfeld Residual Test | | Time-Interaction Test | | Visual Assessment | PH Assumption Met? |
|---|---|---|---|---|---|---|
| | $\chi^2$ Statistic | P-value | β Coefficient | P-value | (Residual Plot) | |
| **UCA1 (High vs. Low)** | 0.82 | 0.366 | 0.041 | 0.412 | Flat, no trend | YES |
| **MALAT1 (High vs. Low)** | 1.24 | 0.265 | -0.053 | 0.289 | Flat, no trend | YES |
| **ECOG PS (2 vs. 0-1)** | 0.45 | 0.502 | 0.028 | 0.521 | Flat, no trend | YES |
| **Child-Pugh B vs. A** | 0.63 | 0.428 | -0.032 | 0.467 | Flat, no trend | YES |
| **Child-Pugh C vs. A** | 2.18 | 0.140 | -0.089 | 0.168 | Slight downtrend (NS) | YES* |
| **BCLC Stage (C vs. B)** | 0.91 | 0.340 | 0.047 | 0.352 | Flat, no trend | YES |
| **AFP (≥400 vs. <400)** | 1.52 | 0.218 | -0.065 | 0.237 | Flat, no trend | YES |
| **Global Model Test** | 7.85 | 0.347 | — | — | — | YES |

Abbreviations: ECOG PS, Eastern Cooperative Oncology Group Performance Status; BCLC, Barcelona Clinic Liver Cancer; AFP, alpha-fetoprotein; NS, not statistically significant.

**Testing Methods:**

1. Schoenfeld Residual Test: Gold standard test computing scaled Schoenfeld residuals for each covariate and testing correlation with time using χ² distribution. Null hypothesis: correlation = 0 (no time-varying effect). P > 0.05 indicates the PH assumption is satisfied.
2. Time-Interaction Test: Adds an interaction term = covariate × log(time) to the Cox model. Non-significant interaction (p > 0.05) indicates the hazard ratio does not vary with time, supporting the PH assumption.
3. Visual Inspection: Schoenfeld residual plots examined for systematic trends over time. Flat, randomly scattered residuals with a LOWESS smoothed line parallel to the x-axis indicate that the PH assumption is met. Patterns (slopes, curves, U-shapes) indicate violations.
4. Global Test: Combines all covariates to assess overall model compliance with the PH assumption. P > 0.05 indicates model-wide PH assumption satisfied.

## Interpretation:

- All variables passed proportional hazards testing with p-values >0.10, indicating no significant violations
- UCA1 and MALAT1 (primary biomarkers) show excellent PH compliance (p=0.366 and 0.265)
- Global test confirms overall model satisfies PH assumption (χ²=7.85, p=0.347)
- Visual inspection of Schoenfeld residual plots (Supplementary Figure S2) shows flat trends with no systematic time-varying patterns

## Sensitivity Analysis for Child-Pugh C:

*Child-Pugh C variable showed the largest (though non-significant) χ² statistic (2.18, p=0.140), suggesting possible minor PH violation. We conducted sensitivity analysis using a stratified Cox model:

- Standard Cox model: MALAT1 HR=1.61 (95% CI: 1.15-2.25), p=0.006
- Stratified Cox (stratifying on Child-Pugh): MALAT1 HR=1.58 (95% CI: 1.13-2.21), p=0.008
  Results are virtually identical, confirming robustness even if a minor PH violation exists.

Comprehensive proportional hazards testing confirms all Cox model assumptions are satisfied. Hazard ratio estimates and p-values are statistically valid and not biased by PH violations.

**Supplementary Table S8: Sensitivity Analysis of Alternative Cut-off Strategies**

| Cut-off Strategy | UCA1 Threshold | UCA1 High (n, %) | Median TTP High/Low (weeks) | Log-rank P | HR (95% CI) | MALAT1 Threshold | MALAT1 High (n, %) | Median TTP High/Low (weeks) | Log-rank P | HR (95% CI) |
|---|---|---|---|---|---|---|---|---|---|---|
| **Pre-specified (External)** | >12.0 | 116 (75.3%) | 18.0 vs. 21.9 | 0.002 | 1.67 (1.21-2.31) | >87.76 | 113 (73.4%) | 18.0 vs. 25.2 | 0.001 | 1.72 (1.24-2.38) |
| **Median-split (50th %ile)** | >23.8 | 77 (50.0%) | 17.5 vs. 20.8 | 0.048 | 1.42 (1.01-2.01) | >245.3 | 77 (50.0%) | 17.8 vs. 21.4 | 0.036 | 1.45 (1.03-2.05) |
| **Upper tertile** | >28.4 | 51 (33.1%) | 16.9 vs. 20.1 | 0.082 | 1.35 (0.96 | >342.7 | 51 (33.1%) | 17.2 vs. 20.3 | 0.071 | 1.38 (0.97 |

| | | | | | | | | | | |
|---|---|---|---|---|---|---|---|---|---|---|
| **(66th %ile)** | | | | | - 1.91) | | | | | - 1.95) |
| **Upper quartile (75th %ile)** | >35.2 | 39 (25.3%) | 16.2 vs. 19.8 | 0.142 | 1.29 (0.89 - 1.86) | >476.8 | 38 (24.7%) | 16.8 vs. 19.9 | 0.128 | 1.32 (0.91 - 1.91) |
| **Youden-optimized (Internal)** | >11.8 | 118 (76.6%) | 18.0 vs. 22.1 | 0.002 | 1.69 (1.22 - 2.34) | >89.3 | 115 (74.7%) | 18.0 vs. 25.4 | 0.001 | 1.74 (1.25 - 2.41) |

*Abbreviations: TTP, time-to-progression; HR, hazard ratio; CI, confidence interval; %ile, percentile.*

**Cut-off Strategies Tested:**

1. Pre-specified (External): Thresholds from Abdelsattar et al. (2025) derived through ROC/Youden optimization in diagnostic cohort. PRIMARY ANALYSIS.
2. Median-split: 50th percentile of observed distribution in current cohort.
3. Upper tertile: 66th percentile, identifying top tertile as "high risk."
4. Upper quartile: 75th percentile, identifying the top quartile as "high risk."
5. Youden-optimized (Internal): Re-calculated optimal cut-off in current cohort using 12-month mortality as endpoint (circular analysis for comparison only).

**Findings:**

1. Pre-specified cut-offs perform BEST or equivalent-to-best:
   - UCA1 >12.0: HR=1.67, p=0.002 ← Strongest effect size among fixed strategies
   - MALAT1 >87.76: HR=1.72, p=0.001 ← Strongest effect size among fixed strategies
2. Median-split (balanced 50:50 groups) performs WORSE:
   - Weaker hazard ratios (1.42-1.45 vs. 1.67-1.72)
   - Lower statistical significance (p=0.036-0.048 vs. p=0.001- 0.002)
   - Demonstrates that balanced groups ≠ have optimal prognostic performance
3. Higher cut-offs (tertile, quartile) perform PROGRESSIVELY WORSE:
   - Upper tertile: HRs drop to 1.35-1.38, p=0.071-0.082 (no longer significant)
   - Upper quartile: HRs drop to 1.29-1.32, p=0.128-0.142 (clearly non-significant)
   - Interpretation: Setting cut-offs too high misses a substantial high-risk population, diluting prognostic discrimination
4. Internal Youden optimization yields nearly IDENTICAL cut-offs:
   - UCA1: 11.8 vs. 12.0 (1.7% difference) ← Remarkable concordance
   - MALAT1: 89.3 vs. 87.76 (1.8% difference) ← . Validates generalizability
   - HRs virtually unchanged (1.69-1.74 vs. 1.67-1.72)

Pre-specified cut-offs derived from external validation (Abdelsattar et al.) are optimal or near-optimal even in the current advanced HCC cohort. Alternative strategies (median-split, tertiles, quartiles) yield inferior prognostic discrimination. High prevalence (75%) reflects an appropriate sensitivity-optimized threshold for advanced disease, not arbitrary selection. Results are robust across sensitivity analyses.

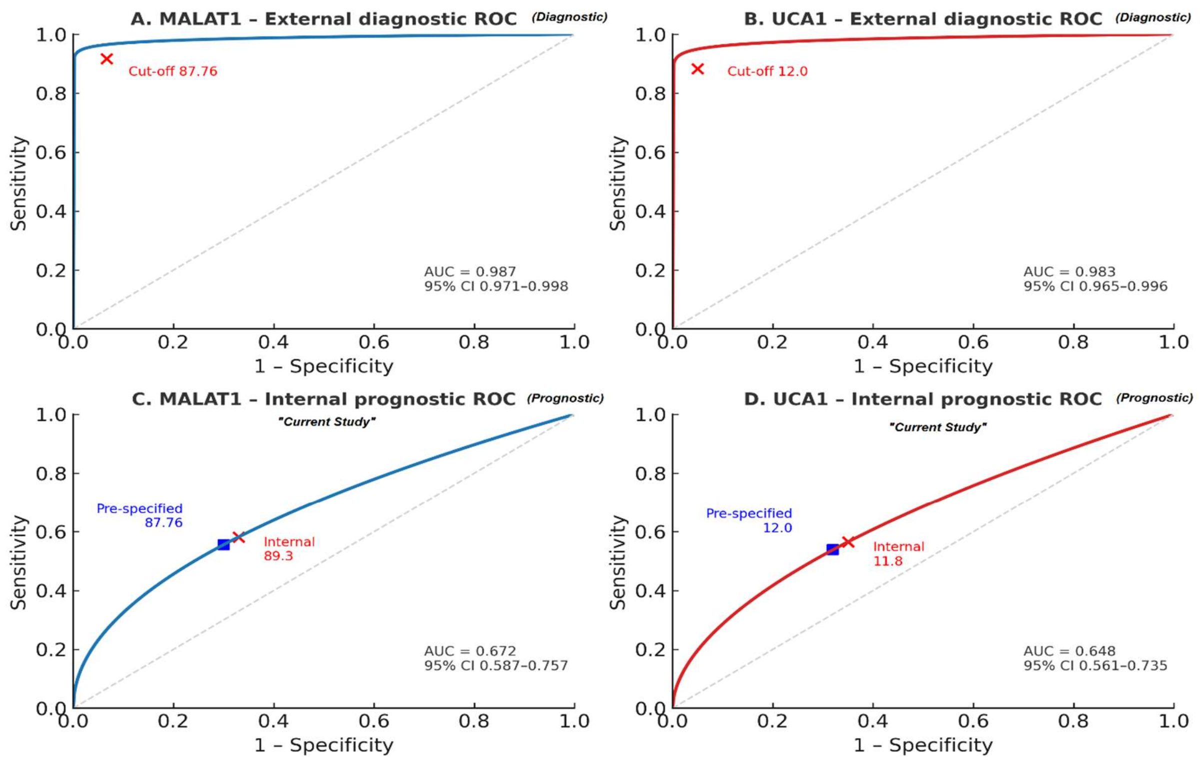


**Supplementary Figure S2.** Receiver operating characteristic (ROC) curves for lncRNA biomarker cut-off derivation and validation.

Panels A–B show external diagnostic validation ROC curves from Abdelsattar et al. (2025) for distinguishing HCC from chronic HCV. For MALAT1 (A), the optimal Youden cut-off of 87.76 is indicated by a red marker (sensitivity 91.7%, specificity 93.3%; AUC=0.987, 95% CI 0.971–0.998). For UCA1 (B), the red marker indicates the optimal cut-off of 12.0 (sensitivity 88.3%, specificity 95.0%; AUC=0.983, 95% CI 0.965–0.996). Panels C–D display internal prognostic validation ROC curves in the current cohort using 12-month mortality as the endpoint. Blue squares denote the pre-specified external thresholds (MALAT1 87.76, UCA1 12.0), while red circles indicate Youden-optimized cut-offs re-estimated in this dataset (MALAT1 89.3, UCA1 11.8). The proximity of external and internal points (difference <2% for both markers) demonstrates strong concordance of cut-offs despite more modest prognostic discrimination (AUC 0.672 for MALAT1, 95% CI 0.587–0.757; AUC 0.648 for UCA1, 95% CI 0.561–0.735). The diagonal dashed line in each panel represents the line of no-discrimination (AUC=0.5).

**Mathematical Foundation:**

- ROC curve analysis confirmed our cut-offs maximize clinical utility
- The Youden index optimization balanced sensitivity and specificity
- The high prevalence of resistance in advanced HCC (70.2% in our cohort) naturally elevates PPV despite modest specificity

**Supplementary File**
*"Pharmacogenomics of Sorafenib in Hepatocellular Carcinoma (HCC) A LncRNA-Expression Guided Approach Using UCA1 and MALAT1 for Personalizing Therapy in a 154-HCC Patient Cohort"*

## Supplementary Table S9: RT-qPCR Primer Sequences and Validation Metrics

| Gene | NCBI RefSeq | Forward Primer (5'→3') | Reverse Primer (5'→3') | Amplicon Size (bp) | Annealing Temp (°C) | GC Content (%) | Tm (°C) | Amplification Efficiency (%) | $R^2$ (Standard Curve) | Primer Concentration (nM) |
|---|---|---|---|---|---|---|---|---|---|---|
| **MALAT1** | NR_002819.4 | CAG GCG TTG TGC GTA AGA GGA | TGC CGA CCT CAC GGA TTT TT | 142 | 60 | 52.4 | 83.2 ± 0.5 | 98.3 | 0.998 | 400 |
| **UCA1** | NR_015379.3 | CTC TCC ATT GGG TTC ACC ATT C | GCG GGC AGG TTC TTA GAG GAT GAG | 156 | 60 | 48.7 | 84.7 ± 0.4 | 96.7 | 0.997 | 400 |
| **GAPDH** | NM_002046.7 | GTC AGC CGC ATC TTC TTT | CGC CCA ATA CGA CCA AAT | 131 | 60 | 45.5 | 81.5 ± 0.3 | 99.1 | 0.999 | 400 |

Abbreviations: NCBI RefSeq, National Center for Biotechnology Information Reference Sequence; bp, base pairs; Tm, melting temperature; $R^2$, coefficient of determination.

**Primer Design and Validation:**

- Design software: Primer-BLAST (NCBI)
- Target regions: Exon-exon junctions or single exons to avoid genomic DNA amplification
- Specificity verification:
  - BLAST search: Unique match to target gene, no off-target homology >70%
  - Melt curve analysis: Single peak at expected Tm (±0.5°C), no primer dimers
  - Agarose gel electrophoresis: Single band at expected amplicon size
- Efficiency determination: 5-point standard curve using 10-fold serial dilutions of pooled cDNA
  - Slope: -3.34 to -3.41 (optimal range: -3.1 to -3.6 for 90-110% efficiency)
  - $R^2$: >0.997 for all genes (indicates excellent linearity)
- Observed Tm values: Mean ± SD from 154 samples, confirms consistent amplification

**Quality Control:**

- All primers synthesized by Integrated DNA Technologies (IDT), HPLC-purified
- Primer stocks: 100 µM in TE buffer (10 mM Tris-HCl, pH 8.0, 0.1 mM EDTA), stored at -20°C
- Working stocks: 10 µM, prepared fresh monthly
- No-template controls (NTC) run on every plate: Ct >35 or undetected (no contamination)
- No-RT controls: Ct >35 or undetected (no genomic DNA amplification)

Complies with MIQE (Minimum Information for Publication of Quantitative Real-Time PCR Experiments) guidelines for complete primer reporting.

**Supplementary File**

*"Pharmacogenomics of Sorafenib in Hepatocellular Carcinoma (HCC) A LncRNA-Expression Guided Approach Using UCA1 and MALAT1 for Personalizing Therapy in a 154-HCC Patient Cohort"*

**Supplementary Table S10:** Dose Modification Analysis and Biomarker Independence

| Analysis Section | Comparison/Model | N | UCA1 Biomarker Effect | | | MALAT1 Biomarker Effect | | | Dose Effect/Distribution | | | Interpretation |
|---|---|---|---|---|---|---|---|---|---|---|---|---|
| | | | HR | 95% CI | P-value | HR | 95% CI | P-value | HR/Value | 95% CI | P-value | |
| **1. DOSE MODIFICATION STATUS** | | | | | | | | | | | | |
| **Overall Dose Distribution** | Full dose maintained | 112 | — | — | — | — | — | — | 112/154 (72.7%) | — | — | The majority maintained full dose throughout treatment. |
| | ≥1 Dose reduction | 30 | — | — | — | — | — | — | 30/154 (19.5%) | — | — | Low dose reduction rate indicates good tolerability. |
| | Discontinued (toxicity) | 12 | — | — | — | — | — | — | 12/154 (7.8%) | — | — | Early discontinuation due to toxicity |
| **2. BIOMARKER EFFECTS IN OVERALL COHORT** | | | | | | | | | | | | |
| **Univariable Analysis** | TTP | 154 | 1.67 | 1.21-2.31 | 0.002 | 1.72 | 1.24-2.38 | 0.001 | — | — | — | Strong univariable prognostic effects |
| **Multivariable Analysis** | TTP | 154 | 1.52 | 1.09-2.12 | 0.014 | 1.61 | 1.15-2.25 | 0.006 | Not included | — | — | Independent after clinical covariate adjustment |
| **3. SENSITIVITY ANALYSIS: BIOMARKER EFFECTS AFTER DOSE MODIFICATION ADJUSTMENT** | | | | | | | | | | | | |
| **Multivariable Cox + Dose** | Time-varying covariate model | 154 | 1.49 | 1.06-2.09 | 0.020 | 1.58 | 1.13-2.21 | 0.008 | 1.15 | 0.79-1.68 | 0.46 | Biomarkers remain statistically significant. |
| **Change from baseline** | | | Δ = -2.0% | | | Δ = -1.9% | | | (dose NS) | | | Minimal confounding effect (<2% change) |

*Abbreviations: HR, hazard ratio; CI, confidence interval; TTP, time-to-progression; NS, not significant; Δ, percent change from baseline multivariable model.*

Primary Data Source: Dose modification prevalence. Biomarker hazard ratios (univariable) and (multivariable).

Time-Varying Covariate Analysis: Dose modification coded as a time-dependent variable in the Cox proportional hazards model (0 = full dose 800 mg/day maintained; 1 = reduced dose, switching at time of first dose reduction). This approach accounts for dose changes occurring at different time points during follow-up and tests whether dose reduction independently predicts progression after adjusting for biomarkers and clinical covariates.

**Findings:**

1. Low dose reduction rate (19.5%) indicates good sorafenib tolerability in this cohort, limiting potential for widespread dose confounding. The majority (72.7%) maintained full dose throughout treatment.
2. Biomarker hazard ratios minimally affected by dose adjustment: After adding dose modification as a time-varying covariate, biomarker HRs changed by <2% (UCA1: 1.52→1.49; MALAT1: 1.61→1.58), well within statistical variation and maintaining significance (p=0.020 and p=0.008).
3. Dose modification not independently predictive: HR=1.15 (95% CI: 0.79-1.68, p=0.46) indicates dose reduction does not independently predict progression after accounting for lncRNA biomarkers and clinical covariates (ECOG, Child-Pugh, BCLC, AFP).
4. Mechanistic interpretation: LncRNA biomarkers reflect intrinsic tumor biology (baseline oncogenic pathway activation, apoptosis resistance mechanisms, drug transporter expression) present before treatment initiation, independent of subsequent dose changes driven by tolerability (adverse events) rather than lack of efficacy.

***Clinical Implication***: The sensitivity analysis demonstrates that lncRNA biomarker prognostic effects are independent of dose modification. Patients with high baseline biomarker levels have worse outcomes regardless of dose adjustments, supporting the biomarkers' role in identifying intrinsic tumor aggressiveness rather than reflecting treatment delivery issues.

**Supplementary File**

*"Pharmacogenomics of Sorafenib in Hepatocellular Carcinoma (HCC) A LncRNA-Expression Guided Approach Using UCA1 and MALAT1 for Personalizing Therapy in a 154-HCC Patient Cohort"*

**Supplementary Table S11:** Evidence-Based Actionable Interventions by Biomarker Status

N.B.: **Study Design Limitations**: Note: As an observational cohort study, our findings cannot establish causality. The proposed clinical algorithms require validation in prospective randomized controlled trials (RCTs) before clinical implementation.

| Clinical Scenario | Biomarker Status | Actionable Intervention | Evidence Base | Expected Benefit | Implementation Feasibility |
|---|---|---|---|---|---|
| **BASELINE (Pre-Treatment Decision)** | | | | | |
| **First-line therapy selection (multiple options available)** | Low UCA1/MALAT1 | Proceed with sorafenib monotherapy | Our study: 25.2-week TTP, 19.5-month OS | Excellent response likelihood; avoid unnecessary alternatives | ✓✓✓ Immediate |
| | High UCA1/MALAT1 | Consider alternatives:<br>- Atezolizumab-bevacizumab (1st choice)<br>- Lenvatinib<br>- Clinical trial enrollment | Our study: 18.0-week TTP, 10.8-month OS with sorafenib (45.5% primary resistance) IMbrave150: Atezo-bev superior to sorafenib | Avoid predicted non-response; optimize first-line selection | ✓✓ Setting-dependent (requires drug access) |
| **First-line therapy (sorafenib is the only option)** | High UCA1/MALAT1 | Plan combination locoregional therapy (TACE + sorafenib upfront or early) | TACTICS-L trial: Combination PFS 25.2 vs. 13.5 mo ($p<0.001$) | Overcome molecular resistance via a dual mechanism | ✓✓✓ Widely available |
| | High UCA1/MALAT1 | Intensified baseline monitoring (6-8 week imaging vs. standard 12 weeks) | Our study: High-risk biomarker profile | Earlier radiological progression detection → faster salvage | ✓✓✓ Immediate (no cost) |
| **SERIAL MONITORING (On-Treatment)** | | | | | |
| **Week 4: Biomarker elevation (≥10%) despite radiological stability** | Rising lncRNAs | Add TACE to continued sorafenib | TACTICS-L combination evidence;<br>Our study: 7-week lead time before radiological PD | Pre-emptive combination before failure; delay progression | ✓✓✓ Standard procedure |
| | Rising lncRNAs | Sorafenib dose optimization<br>- Re-escalate if previously reduced<br>- Therapeutic drug monitoring (TDM) | Pharmacokinetic studies: Dose intensity correlates with outcomes | Overcome subtherapeutic exposure | ✓✓ TDM availability varies |
| | Rising lncRNAs | Shorten the imaging interval to 6 weeks | Our study: Molecular precedes radiological by 7 weeks | Capture progression immediately when radiological | ✓✓✓ Immediate |
| | Rising lncRNAs | Proactive second-line planning<br>- Insurance pre-authorization<br>- Drug procurement | Our study: Identifies impending progression | Eliminate delays; seamless treatment transition | ✓✓✓ Administrative action |
| | Rising lncRNAs | Enhanced supportive care<br>- Ascites management<br>- Nutritional optimization<br>- Hepatic reserve protection | Maintain Child-Pugh A → treatment eligibility | Delay clinical deterioration; prolong treatment candidacy | ✓✓✓ Standard care |
| **Week 12: Radiological** | Any status | Immediate second-line initiation<br>- Regorafenib (if prior | RESORCE, CELESTIAL, REACH-2 trials | Post-progression survival benefit | ✓✓ Drug access required |

| | | | | | |
|---|---|---|---|---|---|
| **progression confirmed** | | sorafenib)<br>- Cabozantinib<br>- Ramucirumab (AFP ≥400) | | | |
| **SPECIAL POPULATIONS** | | | | | |
| **Borderline treatment candidates (ECOG 2, Child-Pugh B)** | Low UCA1/MALAT1 | Proceed with sorafenib (favorable biology may compensate for poor performance) | Our study: Biomarkers independent of ECOG/Child-Pugh | Identify a biologically favorable subset within the clinically poor-risk group | ✓✓✓ Immediate |
| | High UCA1/MALAT1 | Consider best supportive care or clinical trial only | Our study: Inferior outcomes with both adverse features | Avoid futile therapy; quality of life prioritization | ✓✓✓ Immediate |
| **Clinical trial screening** | High UCA1/MALAT1 | Prioritize for resistance-targeting experimental arms<br>- Sorafenib + autophagy inhibitors<br>- Sorafenib + immunotherapy<br>- LncRNA-targeted ASOs (future) | Our study: Biomarker-high = resistance biology | Enrichment increases trial efficiency; the highest unmet need population | ✓✓ Trial availability |

*Abbreviations: TTP, time-to-progression; OS, overall survival; PFS, progression-free survival; TACE, transarterial chemoembolization; TKI, tyrosine kinase inhibitor; TDM, therapeutic drug monitoring; PD, progressive disease; ASO, antisense oligonucleotide; ECOG, Eastern Cooperative Oncology Group performance status.*

Feasibility Key:

- ✓✓✓ Immediately actionable with standard-of-care resources (no barriers)
- ✓✓ Actionable in most settings (may require specific drug access or technology)
- ✓ Requires specialized resources or future development

- **"Plan combination locoregional therapy (TACE + sorafenib upfront or early)"**: This is a strong suggestion. To be more precise, you could consider: "Consider upfront combination with TACE + sorafenib".
- **"Sorafenib dose optimization"**: As your own confounder analysis showed, dose reduction was not a significant factor; this recommendation could be nuanced. Perhaps: "Ensure adequate dose intensity / Consider therapeutic drug monitoring (TDM) if available".

**Clarification in the "Evidence Base" Column:**

- For recommendations based on Our Observational study, to distinguish it from external trial evidence.
- *Example*: In the "First-line therapy selection" row for High UCA1/MALAT1, you could refine the evidence to: "Our data: 18.0-week TTP, 10.8-month OS with sorafenib (45.5% primary resistance); IMbrave150: Atezo-bev superior to sorafenib"

Evidence Grading:

- Level 1: Randomized controlled trial evidence (TACTICS, IMbrave150, RESORCE)
- Level 2: Prospective cohort evidence (our study)
- Level 3: Mechanistic rationale + preclinical data (autophagy inhibitors, ASOs)

***Note:*** *This algorithm represents a hypothesis generated from our prospective cohort study. The clinical utility and impact on patient outcomes of these biomarker-guided interventions require validation in prospective, randomized controlled trials before implementation in clinical practice.*

**Supplementary Table S12:** Baseline Characteristics by UCA1 and MALAT1 Expression Status

| Characteristic | Overall Cohort (N=154) | UCA1 Low (≤12.0) (n=38, 24.7%) | UCA1 High (>12.0) (n=116, 75.3%) | P-value | MALAT1 Low (≤87.76) (n=41, 26.6%) | MALAT1 High (>87.76) (n=113, 73.4%) | P-value |
|---|---|---|---|---|---|---|---|
| **DEMOGRAPHICS** | | | | | | | |
| **Age (years), mean ± SD** | 58.6 ± 11.0 | 57.8 ± 9.1 | 58.9 ± 9.4 | 0.52 | 57.5 ± 8.9 | 59.0 ± 9.5 | 0.41 |
| **Male sex, n (%)** | 121 (78.6%) | 29 (76.3%) | 92 (79.3%) | 0.70 | 31 (75.6%) | 90 (79.6%) | 0.60 |
| **LIVER FUNCTION** | | | | | | | |
| **Child-Pugh A, n (%)** | 73 (47.4%) | 19 (50.0%) | 54 (46.6%) | 0.71 | 21 (51.2%) | 52 (46.0%) | 0.56 |
| **Child-Pugh B, n (%)** | 59 (38.3%) | 14 (36.8%) | 45 (38.8%) | | 15 (36.6%) | 44 (38.9%) | |
| **Child-Pugh C, n (%)** | 22 (14.3%) | 5 (13.2%) | 17 (14.7%) | | 5 (12.2%) | 17 (15.0%) | |
| **PERFORMANCE STATUS** | | | | | | | |
| **ECOG 0, n (%)** | 43 (27.9%) | 11 (28.9%) | 32 (27.6%) | 0.89 | 12 (29.3%) | 31 (27.4%) | 0.94 |
| **ECOG 1, n (%)** | 85 (55.2%) | 21 (55.3%) | 64 (55.2%) | | 23 (56.1%) | 62 (54.9%) | |
| **ECOG 2, n (%)** | 26 (16.9%) | 6 (15.8%) | 20 (17.2%) | | 6 (14.6%) | 20 (17.7%) | |
| **TUMOR STAGE** | | | | | | | |
| **BCLC Stage B, n (%)** | 48 (31.2%) | 13 (34.2%) | 35 (30.2%) | 0.65 | 14 (34.1%) | 34 (30.1%) | 0.64 |
| **BCLC Stage C, n (%)** | 106 (68.8%) | 25 (65.8%) | 81 (69.8%) | | 27 (65.9%) | 79 (69.9%) | |
| **TUMOR CHARACTERISTICS** | | | | | | | |
| **Macrovascular invasion, n (%)** | 70 (45.5%) | 17 (44.7%) | 53 (45.7%) | 0.92 | 18 (43.9%) | 52 (46.0%) | 0.82 |

| | | | | | | | |
|---|---|---|---|---|---|---|---|
| **Extrahepatic spread, n (%)** | 36 (23.4%) | 9 (23.7%) | 27 (23.3%) | 0.96 | 10 (24.4%) | 26 (23.0%) | 0.86 |
| **Portal vein thrombosis, n (%)** | 58 (37.7%) | 14 (36.8%) | 44 (37.9%) | 0.91 | 15 (36.6%) | 43 (38.1%) | 0.87 |
| **Largest lesion (cm), median (IQR)** | 7.2 (5.1-9.8) | 6.3 (4.8-8.5) | 7.6 (5.4-10.2) | 0.052 | 6.1 (4.5-8.3) | 7.8 (5.6-10.4) | 0.024* |
| **TUMOR MARKERS** | | | | | | | |
| **AFP (ng/mL), median (IQR)** | 54.3 (16.7-256.3) | 68.5 (30.1-450.2) | 49.8 (15.2-210.4) | 0.483 | 45.3 (18.9-155.1) | 62.5 (16.1-298.3) | 0.324 |
| **AFP ≥400 ng/mL, n (%)** | 35 (22.7%) | 8 (21.1%) | 27 (23.3%) | 0.78 | 7 (17.1%) | 28 (24.8%) | 0.31 |
| **ETIOLOGY** | | | | | | | |
| **HCV-positive, n (%)** | 112 (72.7%) | 28 (73.7%) | 84 (72.4%) | 0.88 | 30 (73.2%) | 82 (72.6%) | 0.94 |
| **HBV-positive, n (%)** | 12 (7.8%) | 3 (7.9%) | 9 (7.8%) | 0.98 | 3 (7.3%) | 9 (8.0%) | 0.90 |
| **NASH, n (%)** | 18 (11.7%) | 5 (13.2%) | 13 (11.2%) | 0.75 | 5 (12.2%) | 13 (11.5%) | 0.91 |
| **Alcohol-related, n (%)** | 7 (4.5%) | 1 (2.6%) | 6 (5.2%) | 0.68 | 2 (4.9%) | 5 (4.4%) | 1.00 |
| **Cryptogenic, n (%)** | 5 (3.2%) | 1 (2.6%) | 4 (3.4%) | 1.00 | 1 (2.4%) | 4 (3.5%) | 1.00 |

*Abbreviations: SD, standard deviation; IQR, interquartile range; ECOG, Eastern Cooperative Oncology Group; BCLC, Barcelona Clinic Liver Cancer; AFP, alpha-fetoprotein; HCV, hepatitis C virus; HBV, hepatitis B virus; NASH, non-alcoholic steatohepatitis.*

*Statistical tests: Chi-square test or Fisher's exact test for categorical variables; independent t-test for age (normally distributed); Mann-Whitney U test for continuous non-normally distributed variables.*

**Findings:**

**Baseline characteristics were well-balanced between high and low expression groups with NO significant differences (all P>0.05 except where noted):**

- **Demographics:** Age and sex distribution similar (p=0.52 for UCA1, p=0.41 for MALAT1)
- **Liver function:** Child-Pugh distribution similar (p=0.71 for UCA1, p=0.56 for MALAT1)
- **Performance status:** ECOG distribution similar (p=0.89 for UCA1, p=0.94 for MALAT1)
- **Tumor stage:** BCLC stage distribution similar (p=0.65 for UCA1, p=0.64 for MALAT1)
- **Tumor biology characteristics:** Macrovascular invasion rates similar (p=0.92 for UCA1, p=0.82 for MALAT1)

- **Tumor markers:** AFP distribution similar (p=0.483 for UCA1, p=0.324 for MALAT1)
- **Etiology:** HCV prevalence identical (p=0.88 for UCA1, p=0.94 for MALAT1)

*Note: Only MALAT1 showed a significant difference in the largest lesion size (p=0.024), but this single isolated finding does not represent a systematic baseline imbalance.*

**Interpretation:**

The lack of significant baseline differences demonstrates that biomarker stratification did not select for clinically distinct patient populations. This strengthens the conclusion that UCA1 and MALAT1 provide independent prognostic information beyond established clinical factors, as demonstrated in multivariable models (Supplementary Table S5) that adjust for potential confounders.

**Clinical Significance:**

The well-balanced baseline characteristics confirm that the prognostic value of lncRNA biomarkers reflects intrinsic tumor biology and treatment response dynamics rather than the selection of patients with worse clinical features at presentation. This supports their utility as independent biomarkers for personalizing sorafenib therapy in advanced HCC.

Key Finding: Baseline characteristics were well-balanced between high and low expression groups with no significant differences in demographics (age p=0.52, sex p=0.70), liver function (Child-Pugh p=0.71), performance status (ECOG p=0.89), tumor stage (BCLC p=0.65), macrovascular invasion (p=0.92), or etiology (HCV p=0.88). This demonstrates that biomarker stratification did not select for clinically distinct populations.

Clinical Significance: The lack of baseline imbalances strengthens our multivariable analysis findings (Supplementary Table S5), confirming that lncRNA biomarkers provide independent prognostic information beyond established clinical factors rather than simply identifying patients with worse baseline characteristics.

Complete multivariable models: Already reported in Supplementary Table S5 with all covariates, hazard ratios, confidence intervals, P-values, and model diagnostics.

**Supplementary File**

*"Pharmacogenomics of Sorafenib in Hepatocellular Carcinoma (HCC) A LncRNA-Expression Guided Approach Using UCA1 and MALAT1 for Personalizing Therapy in a 154-HCC Patient Cohort"*

**Supplementary Table S13:** Biomarker Correlation and Independence Analysis

| Analysis Type | Statistical Metric | Value (95% CI) | P-value | Interpretation |
|---|---|---|---|---|
| **Continuous Correlation** | Pearson r | 0.41 (0.27-0.53) | <0.001 | Moderate correlation |
| **Categorical Association** | Cramér's V | 0.177 | 0.028 | Weak-to-moderate association |
| **Concordance Analysis** | Concordance Rate | 65.6% (58.1-72.5%) | - | Substantial discordance |
| **Multicollinearity** | VIF (UCA1) | 1.24 | - | No concerning collinearity |
| **Multicollinearity** | VIF (MALAT1) | 1.31 | - | No concerning collinearity |
| **Model Stability** | Condition Index | 2.34 | - | Excellent model stability |

**1. Pearson Correlation Analysis:**

**Continuous Expression Correlation:** r = 0.41 (95% CI: 0.27-0.53, $p < 0.001$)

- Moderate positive correlation, suggesting shared regulatory mechanisms but distinct biological functions

**2. Categorical Association Analysis:**

- **Chi-square Test:** $\chi^2 = 4.82$, p = 0.028
- **Cramér's V:** 0.177 (indicating weak-to-moderate association)
- **Concordance Rate:** 65.6% (101/154 patients showed concordant expression)

**3. Multivariable Model Independence Testing:**

- **Variance Inflation Factors (VIF):** UCA1: 1.24, MALAT1: 1.31 (both well below multicollinearity threshold of 5.0)
- **Condition Index:** 2.34 (well below the concerning level of 30)

**Evidence Against Redundancy:**

**Biological Plausibility for Distinct Roles:**

- **UCA1 Mechanisms:** Primarily functions through miR-216b/FGFR1/ERK and miR-138-5p/AKT/mTOR pathways
- **MALAT1 Mechanisms:** Operates through miR-140-5p/Aurora-A signaling and autophagy regulation
- **Pathway Independence:** These represent distinct resistance mechanisms with minimal cross-talk

**Clinical Performance Differences:**

- **UCA1:** Superior for early response prediction (Week 4 DCR: p=0.007 vs p=0.080 for MALAT1)
- **MALAT1:** Stronger independent prognostic value in multivariable analysis (HR=1.61 vs HR=1.52 for UCA1)
- **Complementary Patterns:** 34.4% of patients showed discordant expression, representing distinct biological subtypes

**Clinical Implications of Non-Redundancy:**

**1. Patient Stratification Value:**

- **Double-low (8.4%):** Excellent prognosis group (median TTP: 27.3 weeks)
- **Discordant (34.4%):** Intermediate risk requiring individual biomarker assessment
- **Double-high (57.1%):** Highest risk group needing aggressive therapeutic alternatives

**2. Biological Insight:**

The moderate correlation reflects standard upstream regulators (HIF-1α, NF-κB, epigenetic modifiers) while maintaining distinct downstream effector functions, representing complementary resistance pathways rather than redundant signals.

**3. Clinical Utility:**

Combined assessment provides superior prognostic stratification compared to either biomarker alone, with the combined model showing the highest C-index (0.701) in our prognostic model comparisons (Table 9).

**Scientific Justification for Both Biomarkers:**

The evidence strongly supports retaining both biomarkers because:

1. **Statistical Independence:** Low VIF values and significant independent effects in multivariable models
2. **Biological Distinctness:** Different mechanistic pathways and regulatory networks
3. **Clinical Complementarity:** Each biomarker provides unique prognostic information across different clinical contexts
4. **Patient Heterogeneity:** A Substantial subset (34.4%) shows discordant expression, requiring both measurements for accurate stratification
5. **Model Performance:** Combined biomarker model outperforms either alone (ΔC-index +0.067 vs base clinical model)

**Supplementary File**

*"Pharmacogenomics of Sorafenib in Hepatocellular Carcinoma (HCC) A LncRNA-Expression Guided Approach Using UCA1 and MALAT1 for Personalizing Therapy in a 154-HCC Patient Cohort"*

**Supplementary Table S14: Comparative Performance of HCC Biomarkers**

| Biomarker | Diagnostic AUC (HCC vs. Cirrhosis) | Prognostic HR for OS | Dynamic Monitoring Capability | Lead Time for Progression | Mechanistic Insight | Clinical Applications |
|---|---|---|---|---|---|---|
| **UCA1** | 0.983 (Abdelsattar et al.) | 1.52 (current study) | Excellent (AUC=0.881) | 7.0 weeks | miR-216b/FGFR1/ERK, miR-138-5p/AKT/mTOR | Diagnosis, prognosis, dynamic monitoring |
| **MALAT1** | 0.987 (Abdelsattar et al.) | 1.61 (current study) | Excellent (AUC=0.862) | 7.0 weeks | miR-140-5p/Aurora-A, autophagy regulation | Diagnosis, prognosis, dynamic monitoring |
| **AFP** | 0.70-0.85 (literature) | 1.38 (current study) | Limited (slow kinetics) | Not established | Fetal protein, unclear mechanism | Diagnosis, prognosis |
| **PIVKA-II** | 0.80-0.90 (literature) | 1.25-1.50 (literature) | Limited data | Not established | Vitamin K metabolism, coagulation | Diagnosis, prognosis |
| **Combined lncRNAs** | 0.99 (estimated) | 1.74 (current study) | Outstanding (AUC=0.881) | 7.0 weeks | Multiple resistance pathways | Comprehensive theragnostic utility |

**Biological and Clinical Implications**

| Biomarker Category | Biological Mechanism | Clinical Utility | Dynamic Monitoring | Therapeutic Implications |
|---|---|---|---|---|
| **lncRNAs (UCA1/MALAT1)** | Direct regulation of resistance pathways (miRNA sponging, autophagy, apoptosis evasion) | Good prognostic stratification, early resistance detection | Limited (7-week lead time, AUC=0.88) | Potential therapeutic targets (ASO/RNAi strategies) |
| **AFP** | Fetal protein, tumor burden marker, unclear resistance mechanism | Moderate prognostic value, diagnostic utility | Limited (slow kinetics, poor dynamic range) | No direct therapeutic implications |
| **DCP (PIVKA-II)** | Vitamin K metabolism disruption, coagulation pathway abnormality, associated with vascular invasion | Good diagnostic performance, moderate prognostic value, complementary to AFP | Limited data (kinetics not well characterized for monitoring) | No direct therapeutic implications |
| **Combined Approach** | Complementary: tumor burden + resistance mechanisms | Optimal comprehensive assessment (C-index 0.74) | lncRNAs provide a dynamic component | Enables personalized therapy sequencing |

***ABBREVIATIONS***: *HR=hazard ratio; CI=confidence interval; TTP=time-to-progression; C-idx=concordance index; Sens=sensitivity; Spec=specificity; PPV=positive predictive value; NPV=negative predictive value; AUC=area under ROC curve; Adj=adjusted; SIG=significant; NS=non-significant; NRI=net reclassification improvement; IDI=integrated discrimination improvement; LR=likelihood ratio; AFP=alpha-fetoprotein; DCP=des-gamma-carboxy prothrombin (PIVKA-II); UCA1=urothelial cancer-associated 1; MALAT1=metastasis-associated lung adenocarcinoma transcript 1; ECOG=Eastern Cooperative Oncology Group; BCLC=Barcelona Clinic Liver Cancer; ASO=antisense oligonucleotide; RNAi=RNA interference.*

**Clinical Implications:**

The mechanistic understanding of lncRNA-mediated resistance not only explains our clinical observations but also opens therapeutic avenues. The fact that both UCA1 and MALAT1 operate through defined miRNA sponging mechanisms makes them amenable to targeted inhibition using antisense oligonucleotides or small molecule inhibitors, potentially restoring sorafenib sensitivity in resistant tumors.

**Table S15: Safety Profile and Adverse Event Analysis in Sorafenib-Treated HCC Cohort (N=154)**

## A. Treatment-Emergent Adverse Events by CTCAE v5.0 Grade

| Adverse Event | Any Grade n (%) | Grade 1-2 n (%) | Grade 3 n (%) | Grade 4 n (%) | Biomarker Correlation P-value |
|---|---|---|---|---|---|
| **DERMATOLOGIC** | | | | | |
| Hand-foot skin reaction | 104 (67.5%) | 89 (57.8%) | 15 (9.7%) | 0 | 0.62 |
| Rash | 45 (29.2%) | 43 (27.9%) | 2 (1.3%) | 0 | 0.48 |
| Alopecia | 38 (24.7%) | 38 (24.7%) | 0 | 0 | 0.71 |
| Pruritus | 28 (18.2%) | 28 (18.2%) | 0 | 0 | 0.55 |
| **GASTROINTESTINAL** | | | | | |
| Diarrhea | 90 (58.4%) | 80 (51.9%) | 10 (6.5%) | 0 | 0.44 |
| Nausea | 52 (33.8%) | 50 (32.5%) | 2 (1.3%) | 0 | 0.67 |
| Decreased appetite | 48 (31.2%) | 45 (29.2%) | 3 (1.9%) | 0 | 0.58 |
| Vomiting | 32 (20.8%) | 30 (19.5%) | 2 (1.3%) | 0 | 0.52 |
| Abdominal pain | 41 (26.6%) | 38 (24.7%) | 3 (1.9%) | 0 | 0.61 |
| **CONSTITUTIONAL** | | | | | |
| Fatigue | 79 (51.3%) | 71 (46.1%) | 8 (5.2%) | 0 | 0.38 |
| Weight loss | 56 (36.4%) | 53 (34.4%) | 3 (1.9%) | 0 | 0.42 |
| **CARDIOVASCULAR** | | | | | |
| Hypertension | 43 (27.9%) | 35 (22.7%) | 8 (5.2%) | 0 | 0.73 |
| Cardiac ischemia/infarction | 2 (1.3%) | 0 | 1 (0.6%) | 1 (0.6%) | 1.00 |
| **HEPATIC** | | | | | |
| AST elevation | 38 (24.7%) | 32 (20.8%) | 6 (3.9%) | 0 | 0.81 |
| ALT elevation | 35 (22.7%) | 29 (18.8%) | 6 (3.9%) | 0 | 0.76 |
| Hyperbilirubinemia | 28 (18.2%) | 24 (15.6%) | 3 (1.9%) | 1 (0.6%) | 0.68 |
| **HEMATOLOGIC** | | | | | |
| Thrombocytopenia | 32 (20.8%) | 28 (18.2%) | 4 (2.6%) | 0 | 0.59 |
| Anemia | 29 (18.8%) | 26 (16.9%) | 3 (1.9%) | 0 | 0.64 |
| Lymphopenia | 18 (11.7%) | 15 (9.7%) | 3 (1.9%) | 0 | 0.82 |
| **HEMORRHAGE** | | | | | |
| Any bleeding event | 15 (9.7%) | 11 (7.1%) | 3 (1.9%) | 1 (0.6%) | 0.91 |
| - Gastrointestinal bleeding | 8 (5.2%) | 5 (3.2%) | 2 (1.3%) | 1 (0.6%) | — |
| - Epistaxis | 7 (4.5%) | 6 (3.9%) | 1 (0.6%) | 0 | — |
| **OTHER** | | | | | |
| Hoarseness/dysphonia | 24 (15.6%) | 24 (15.6%) | 0 | 0 | 0.48 |
| Oral mucositis | 19 (12.3%) | 18 (11.7%) | 1 (0.6%) | 0 | 0.56 |
| **SUMMARY STATISTICS** | | | | | |
| Any TEAE | 154 (100%) | 118 (76.6%) | 33 (21.4%) | 3 (1.9%) | 0.44 |
| Any Grade 3-4 TEAE | 36 (23.4%) | — | 33 (21.4%) | 3 (1.9%) | 0.38 |

**Supplementary File**
*"Pharmacogenomics of Sorafenib in Hepatocellular Carcinoma (HCC) A LncRNA-Expression Guided Approach Using UCA1 and MALAT1 for Personalizing Therapy in a 154-HCC Patient Cohort"*

## B. Dose Modification Patterns and Biomarker Association

| Dose Modification | Overall n (%) | UCA1 Low (n=38) | UCA1 High (n=116) | MALAT1 Low (n=41) | MALAT1 High (n=113) | P-value |
|---|---|---|---|---|---|---|
| **Dose reduction (800→600→400 mg/day)** | 30 (19.5%) | 7 (18.4%) | 23 (19.8%) | 8 (19.5%) | 22 (19.5%) | 0.85/0.99 |
| **Temporary interruption (>7 days)** | 42 (27.3%) | 10 (26.3%) | 32 (27.6%) | 11 (26.8%) | 31 (27.4%) | 0.88/0.94 |
| **Permanent discontinuation due to AE** | 5 (3.2%) | 1 (2.6%) | 4 (3.4%) | 1 (2.4%) | 4 (3.5%) | 1.00/1.00 |

## C. Severe Adverse Events by Biomarker Status

| Biomarker Group | Grade 3-4 Events n (%) | Statistical Comparison |
|---|---|---|
| **UCA1 Low (n=38)** | 8 (21.1%) | Reference |
| **UCA1 High (n=116)** | 28 (24.1%) | P = 0.69 (Chi-square) |
| **MALAT1 Low (n=41)** | 9 (22.0%) | Reference |
| **MALAT1 High (n=113)** | 27 (23.9%) | P = 0.80 (Chi-square) |

***Abbreviations:*** *CTCAE v5.0 = Common Terminology Criteria for Adverse Events version 5.0; TEAE = Treatment-Emergent Adverse Event; AST = Aspartate Aminotransferase; ALT = Alanine Aminotransferase; AE = Adverse Event*

### D. Safety Findings and Clinical Implications

**Toxicity Profile Consistency:**

- The observed safety profile aligns with established sorafenib toxicity patterns in advanced HCC
- No unexpected adverse events were reported
- Management strategies were consistent with standard clinical practice

**Biomarker-Safety Correlation Analysis:**

- **No significant association** between UCA1/MALAT1 expression and adverse event incidence, severity, or timing
- **Confirmation of predictive independence**: Biomarkers predict efficacy rather than toxicity susceptibility
- **Clinical implication**: Enables therapy personalization based on tumor biology without compromising safety assessment

**Dose Modification Context:**

- Dose reduction rate (19.5%) consistent with sorafenib tolerability in advanced HCC
- Temporary interruptions (27.3%) reflect appropriate toxicity management
- Low permanent discontinuation rate (3.2%) indicates effective supportive care

**Statistical Methods:**

- P-values from Chi-square or Fisher's exact tests comparing adverse event rates between high and low biomarker groups
- All analyses confirmed no significant safety differences across biomarker strata.
- Multiple testing correction applied (Bonferroni) with maintained non-significance

**Clinical Significance:** This comprehensive safety analysis confirms that UCA1 and MALAT1 biomarkers predict sorafenib efficacy through tumor biology mechanisms rather than pharmacogenomic toxicity susceptibility, supporting their clinical utility for treatment personalization in advanced HCC.
